# Prevalence of MASLD and fibrosis in Turkey: Results from a multicenter study of at-risk populations

**DOI:** 10.1371/journal.pone.0341214

**Published:** 2026-02-12

**Authors:** Gediz Dogay Us, Francesco Innocenti, Ozgur Muhammet Koc, Volkan Demirhan Yumuk, Zeynep Banu Gungor, Ger H. Koek

**Affiliations:** 1 School of Nutrition and Translational Research in Metabolism, Maastricht University, Maastricht, the Netherlands; 2 Department of Gastroenterology, Pax Clinic, Istanbul, Turkey; 3 Division Endocrinology, Metabolism and Diabetes, Cerrahpaşa Medical Faculty, Istanbul University-Cerrahpasa, Istanbul, Turkey; 4 Department of Methodology and Statistics, CAPHRI Care and Public Health Research Institute, Maastricht University, Maastricht, the Netherlands; 5 Department of Gastroenterology and hepatology, Maastricht University Medical Center, Maastricht, the Netherlands; 6 Department of Medical Biochemistry, Cerrahpaşa Medical Faculty, Istanbul University-Cerrahpasa, Istanbul, Turkey; Kaohsiung Medical University, TAIWAN

## Abstract

**Background:**

Turkey represents a high-risk setting for metabolic dysfunction-associated steatotic liver disease (MASLD), with national obesity and diabetes rates of 32% and 15%, respectively. Yet, no prior studies have systematically assessed MASLD and fibrosis prevalence and the independent contribution of these metabolic risk factors in at-risk Turkish populations exhibiting cardiometabolic risks.

**Methods:**

1,039 adults were enrolled in a multicenter cross-sectional study conducted between 2022 and 2024. All participants presented with at least one of the current MASLD diagnostic criteria. Standardized clinical assessments were performed. Individuals with excessive alcohol consumption (<30 grams/day for men and <20 grams/day for women) were excluded. Steatosis and fibrosis were evaluated using controlled attenuation parameter (CAP) and liver stiffness measurement (LSM) via vibration-controlled transient elastography. MASLD was defined as CAP ≥ 248 dB/m, and significant fibrosis as LSM ≥ 8 kPa. Multivariable logistic regression analyses were used to identify factors associated with MASLD and fibrosis.

**Results:**

The mean age of the study population was 51.7 ± 13.1 years, and 49.4% were male. The mean body mass index was 30.54 ± 5.85 kg/m^2^. Obesity and central obesity were seen in 48.5% and 75.1% of the subjects, respectively. MASLD, significant fibrosis (>F2) and advanced fibrosis (>F3) prevalence were 57.5% (95% CI: 54.4%–60.4%), 7.6% (95% CI: 6.1%–9.4%) and 2.6% (95% CI: 1.8%–3.8%) respectively. Multivariable logistic regression of MASLD revealed a significant association with female sex (OR=0. 548; 95% CI: 0.391, 0.770), obesity (OR=2.208; 95% CI: 1.535, 3.177), insulin resistance (OR=2.09; 95% CI: 1.528, 2.881), metabolic syndrome (OR=2.436; 95% CI: 1.614, 3.679) and central obesity (OR=2.816; 95% CI: 1.725, 4.595). Multivariable logistic regression of fibrosis demonstrated a significant association with high income (OR=0.256; 95% CI: 0.069, 0.948), obesity (OR=4.845; 95% CI: 2.540, 9.242), diabetes, (OR=2.172; 95% CI: 1.306, 3.610), insulin resistance OR=4.205; 95% CI: 2.144, 8.246) and metabolic syndrome (OR=2.053; 95% CI: 1.042, 4.045).

**Conclusion:**

MASLD is prevalent in more than half of the at-risk population studied. Male sex, obesity, metabolic syndrome, insulin resistance and central obesity are significant risk factors associated with it. Despite the high MASLD prevalence, significant fibrosis is less prevalent and is associated with obesity, metabolic syndrome, insulin resistance and diabetes. Further research is warranted in this population.

**Study Registration:**

ClinicalTrials.gov, ID: NCT05194553.

## 1. Introduction

Metabolic dysfunction-associated steatotic liver disease (MASLD) has emerged as a growing global epidemic, with a prevalence estimated between 30–38% [1,2]. The burden is particularly high in South America and the Middle East, where MASLD represents a significant public health concern [3]. In parallel, the clinical relevance of MASLD in Turkey has become increasingly evident, given the rising rates of associated cardiometabolic comorbidities in the country.

Despite its public health implications, the prevalence and characteristics of MASLD in Turkey remain poorly defined, largely due to inconsistencies in study design, methodology, and diagnostic criteria across existing research. Reported prevalence rates in ostensibly healthy populations vary widely, from 10.6% to 65.6%, although many of these studies are limited by suboptimal diagnostic modalities [4,5], retrospective designs [6–8], or small sample sizes [9,10]. A retrospective study with a larger cohort (n = 113.239) estimated a prevalence of 48.3% in the general population [11]. Given that MASLD is strongly associated with cardiometabolic risk factors, higher prevalence rates would be expected in at-risk populations with metabolic disorders. However, only a limited number of studies have investigated this in Turkish at-risk groups. Among patients with diabetes, retrospective reports of MASLD defined by ultrasound has reported prevalence rates ranging from 69.9% to 71.9% [12,13]. Small-scale studies among subjects with diabetes [14] and obesity reported a high prevalence of 94.3% and 88.7% [15], respectively, though MASLD risk factors remain uninvestigated in both. Other key metabolic drivers of MASLD—such as insulin resistance, abdominal obesity, and metabolic syndrome (MetS)—remain unexplored in Turkish populations. According to current MASLD diagnostic criteria, individuals with abdominal obesity, dyslipidemia, impaired glucose metabolism, or elevated blood pressure are considered at risk for MASLD. The independent contribution of these metabolic factors to disease severity in at-risk Turkish cohorts remains to be elucidated.

Therefore, we aimed to investigate the prevalence and risk factors of MASLD and significant fibrosis in at-risk populations to provide a more comprehensive understanding of MASLD burden and its metabolic correlates in this specific population.

## 2. Materials and methods

We conducted a cross-sectional study involving adult participants who visited Istanbul University-Cerrahpasa Hospital obesity division of the endocrinology clinic and an affiliated secondary care internal medicine outpatient center in Istanbul for regular follow-up or initial assessment. The study was originally designed and registered as a single-center study to be conducted at the outpatient center. Subsequently, the second center employing the same inclusion and exclusion criteria was incorporated to decrease selection bias and improve the representativeness of the sample, particularly with respect to socioeconomic and health status. Participant enrollment occurred at both sites between February 2022 and December 2024. The study received ethical approval from the relevant institutional review boards (Istanbul University-Cerrahpasa Clinical Research Ethics Committee, Ref. 2023/134; Istanbul Health Sciences University Clinical Research Ethics Committee, Ref. 2021/224) and was conducted in accordance with the Declaration of Helsinki. Written informed consent was obtained from all participants. The study was prospectively registered with ClinicalTrials.gov (ID: NCT05194553).

### 2.1. Study population

Eligible participants were adults aged 18–80 years who met at least one cardiometabolic risk factor required for MASLD diagnosis (central obesity, impaired glucose metabolism, hypertriglyceridemia, low HDL cholesterol, increased blood pressure) at the time of enrollment, could provide informed consent, and were able to speak, read, and write in Turkish. Exclusion criteria included self-reported excessive alcohol intake (<30 grams per day for men and <20 grams per day for women), a history of hepatotoxic drug use, other known etiological factors of chronic liver disease, prior diagnosis of liver cancer, and current pregnancy or breastfeeding. A comprehensive list of exclusion criteria is provided in S1 Table.

### 2.2. Assessments

After providing written informed consent, participants attended an in-person study visit, which included anthropometric measurements, blood sample collection, MASLD assessment, and completion of self-administered questionnaires on demographics and lifestyle behaviors.

#### 2.2.1. Demographics and clinical data.

A structured questionnaire was used to gather information on marital status, employment status, household size, household monthly income, highest educational attainment and lifestyle habits. Household income levels were categorized into five groups based on quintiles reported annually by the Turkish Statistical Institute [16]. Marital status, income level and education level of the study cohort were compared to reported national statistics to assess representativeness of the sample. Smoking and alcohol consumption were self-reported by participants. Information on age, underlying health conditions and current medication use was extracted from participants’ medical records. The presence of diabetes was determined according to American Diabetes Association criteria by the presence of any 1 of the following 3 conditions: (1) self-reported medical history of diabetes; (2) use of oral hypoglycemic agents or insulin use; and (3) a fasting glucose level greater than or equal to 126 mg/dL or hemoglobin A1c level of 6.5%. The presence of MetS was determined according to International Diabetes Federation criteria.

#### 2.2.2. Anthropometrics.

Body weight was measured to the nearest 0.05 kg using a calibrated electronic scale with bioelectrical impedance analysis (Tanita, Japan), with participants wearing light clothing, barefoot, and in a fasting state. Height was recorded to the nearest 0.1 cm. Body mass index (BMI) was calculated as weight in kilograms divided by the square of height in meters (kg/m^2^). Waist circumference (WC) was measured with a tape measure. Participants with high waist circumference (WC > 80 cm in females and WC > 94 cm in males) were included in the category of central obesity.

#### 2.2.3. Biochemical.

A 10-h fasting blood draw was taken by a registered nurse. Liver enzymes (aspartate aminotransferase (AST), alanine aminotransferase (ALT), gamma-glutamyl transferase (GGT), fasting blood glucose (FBG), total cholesterol (TC), triglycerides (TAG), high-density lipoprotein (HDL)-cholesterol, and low-density lipoprotein (LDL)-cholesterol levels were analyzed using a biochemistry analyzer (Cobas, Roche Diagnostics, Basel, Switzerland) and commercial Roche diagnostics kits. Fasting insulin level was measured using the electrochemiluminescence immunoassay method (Cobas, Roche Diagnostics, Basel, Switzerland). Fasting insulin and glucose concentrations were used to calculate insulin resistance from the HOMA-IR model, with a value of ≥2.5 considered indicative of insulin resistance.

#### 2.2.4. Liver assessments.

Hepatic steatosis and fibrosis were evaluated using vibration-controlled transient elastography (VCTE) with the controlled attenuation parameter (CAP), performed via FibroScan equipped with both M and XL probes. The M probe was used as the default; however, the operator switched to the XL probe when recommended by the device. Examinations were considered valid if participants had fasted for at least three hours, yielded at least 10 valid liver stiffness measurements (LSM), and had an interquartile range to median ratio (IQR/median) below 30%. Steatosis was defined as CAP ≥ 248 dB/m, and significant fibrosis was defined as LSM ≥ 8 kPa in accordance with current clinical guidelines [1] and supported by previous studies [17–20]. MASLD was defined as presence of liver steatosis assessed by CAP, together with at least one of the five cardiometabolic risk factors defined [1]. Steatosis grades were classified using the following controlled attenuation parameter (CAP) cutoff values: S0 (no steatosis) for CAP < 248 dB/m; S1 (mild steatosis) for CAP 248 to <268 dB/m; S2 (moderate steatosis) for CAP 268 to <280 dB/m; and S3 (severe steatosis) for CAP ≥ 280 dB/m [21]. Fibrosis staging was based on liver stiffness measurement (LSM) thresholds: F0–F1 for LSM < 8 kPa; significant fibrosis (F2) for LSM 8–9.6 kPa; advanced fibrosis (F3) if ≥9.7–13.5 kPa, and cirrhosis (F4) if ≥13.6 kPa, according to a with current clinical guidelines [1] and a previous landmark study [22].

### 2.3. Statistical analysis

#### 2.3.1. Sample size calculation.

The sample size was calculated to enable precise estimation of MASLD prevalence, the primary objective of the study. Assuming a true prevalence of 50%, a sample of 384 participants would yield a 95% confidence interval with a margin of error of 5%. For true prevalence values lower or higher than 50%, the margin of error would be even smaller. After the protocol registration, the inclusion of a second center expanded recruitment capacity, making it possible to enroll over 1,000 participants. A sample size of 1,000 corresponds to a margin of error of 3.1% at a true prevalence of 50%, and an even smaller margin of error for prevalences above or below this value.

#### 2.3.2. Data analysis.

Continuous variables with normal distribution were expressed as mean ± SD and assessed by unpaired Student’s t-test to assess between-group (MASLD vs non-MASLD, Fibrosis vs. non-Fibrosis) mean differences. Categorical variables were expressed as frequencies with percentages, and chi square test was used to assess differences between groups. For all variables considered, there were no missing values among participants included in the study. Multivariable logistic regressions with backward elimination based on likelihood ratio tests were performed to identify the risk factors of MASLD and fibrosis, including sex, age, marital status, alcohol, smoking, income level, years of education, presence of obesity, type 2 diabetes, insulin resistance, hypertension, dyslipidemia, metabolic syndrome, central obesity and research site variables in the first model. Risk factors were selected *a-priori* based on previous literature and clinical relevance. Those factors with a p-value >0.157 were removed during the backward selection process [23]. The following variables were excluded from the selection process and included as covariates in all multivariable models irrespective of their p-values: age and sex, as they were considered potential confounders for diabetes, insulin resistance, metabolic syndrome, hypertension and dyslipidemia, and research site, which was treated as fixed effects to account for the hierarchical structure of the data, i.e., patients nested within centres [24]. The assumptions of logistic regression were assessed as follows: linearity of continuous predictors was evaluated by testing for quadratic effects; multicollinearity was identified using variance inflation factors greater than 10; and influential outliers were detected using Cook’s distance values above 1. All statistical analyses were performed using SPSS v30.0 (IBM, USA), and p values < 0.05 were considered statistically significant.

## 3. Results

### 3.1. Patient characteristics

**Fig 1:**
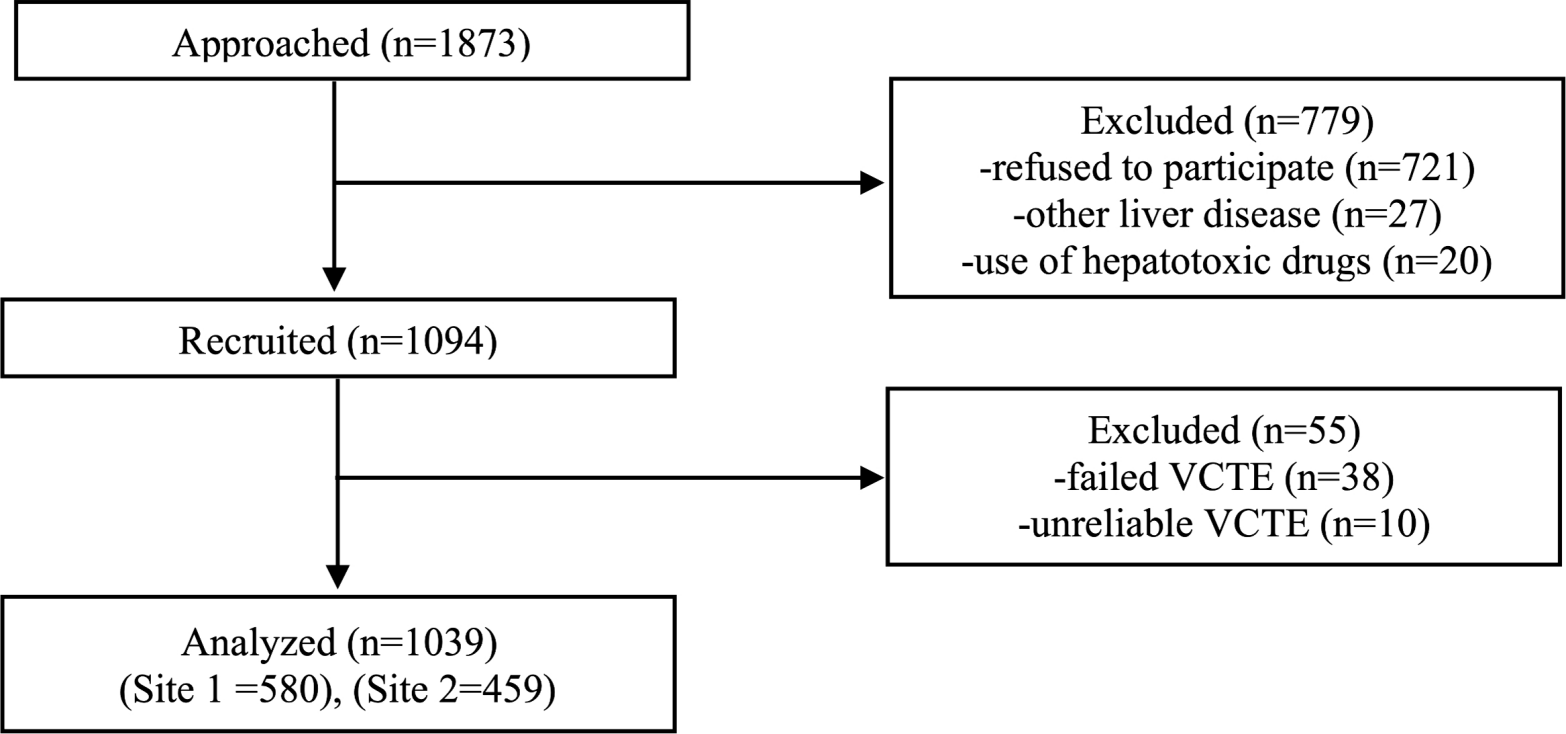
Study flowchart.

A total of 1,094 individuals were recruited for this study, of whom 1,039 were included in the final analysis (Fig 1). The mean age of the study population was 51.7 ± 13.1 years, and 49.4% (n = 513) were male. The mean body mass index (BMI) was 30.54 ± 5.85 kg/m². Obesity and central obesity were prevalent in 48.5% (n = 504) and 75.1% (n = 780) of the subjects, respectively. Detailed characteristics of the study population are presented in Table 1. The demographic profile of the study cohort was broadly representative of the general Turkish population, with the exception of educational attainment, which was higher among study participants (S2 Table). Differences in socioeconomic and health status of the participants in two research sites are provided in S3 Table.

**Table 1:** Characteristics of patients with and without MASLD based on transient elastography.

	All (n = 1039)	MASLD (n = 597)	No-MASLD (n = 442)	p
**Demographics**				
Age (years)	51.7 ± 13.1	52.9 ± 12.2	50.1 ± 13.9	<0.001
Male (%)	513 (49.4)	307 (51.4)	206 (46.6)	NS
Education (years)	12.1 ± 4.7	11.5 ± 4.9	13.0 ± 4.3	<0.001
*• Primary school (%)*	193 (18.6)	142 (23.8)	51 (11.5)	<0.001
*• Secondary school (%)*	94 (9.0)	57 (9.5)	37 (8.4)	<0.001
*• High School (%)*	285 (27.4)	155 (26.0)	130 (29.4)	<0.001
*• University or higher (%)*	467 (45.0)	243 (40.7)	224 (50.7)	<0.001
Married (%)	761 (73.2)	449 (75.2)	312 (70.6)	NS
Very low & Low Income (%)	147 (14.0)	90 (15.1)	57 (12.9)	NS
Medium & Medium High Income (%)	565 (54.4)	325 (54.4)	240 (54.3)	NS
High Income (%)	327 (31.5)	182 (30.5)	145 (32.8)	NS
**Anthropometrics**				
Weight (kg)	86.0 ± 17.5	91.9 ± 16.6	78.1 ± 15.3	<0.001
BMI (kg/m^2^)	30.5 ± 5.9	32.7 ± 5.6	27.6 ± 4.9	<0.001
Central obesity (%)	780 (75.1)	546 (91.5)	234 (52.9)	<0.001
**Lifestyle**				
Drinking (%)	410 (39.5)	201 (33.7)	209 (47.3)	<0.001
Alcohol consumption (g/week)	43.6 ± 79.1	41.6 ± 84.3	46.3 ± 71.6	NS
Smoking (%)	204 (29.3)	166 (27.8)	138 (31.2)	NS
**Biochemical and Imaging Assessments**				
CAP (db/m)	260.1 ± 54.4	297.5 ± 36.1	209.4 ± 27.0	<0.001
LSM (kPa)	5.5 ± 2.1	6.0 ± 2.2	4.8 ± 1.8	<0.001
SSM (kPa) (n = 551)	26.4 ± 12.0	28.2 ± 12.7	24.9 ± 11.2	0.001
Heart rate (bpm)	70.8 ± 9.7	71.6 ± 9.6	69.7 ± 9.8	0.002
HgA1c (%) (n = 987)	5.9 ± 1.2	6.1 ± 1.3	5.6 ± 1.0	<0.001
FBG (mg/dL)	101.3 ± 38.7	106.6 ± 42.7	94.2 ± 31.2	<0.001
HOMA-IR	3.9 ± 4.0	4.7 ± 4.2	2.7 ± 3.5	<0.001
ALT (IU/L)	24.2 ± 17.4	25.9 ± 18.3	22.1 ± 15.9	<0.001
AST (IU/L)	21.0 ± 12.0	21.6 ± 13.3	20.1 ± 10.0	0.034
GGT (IU/L)	28.4 ± 31.2	31.7 ± 32.2	23.8 ± 29.2	<0.001
TC (mg/dL)	196.0 ± 47.5	195.1 ± 48.1	197.3 ± 46.7	NS
LDL (mg/dL)	116.6 ± 41.6	116.4 ± 43.3	116.8 ± 39.1	NS
HDL (mg/dL)	48.4 ± 14.5	45.1 ± 13.7	53.0 ± 14.6	<0.001
TAG (mg/dL)	153.6 ± 101.9	171.1 ± 109.7	130.0 ± 84.7	<0.001
Ferritin	125.3 ± 150.5	126.1 ± 140.8	124.3 ± 161.4	NS
**Non-invasive indices**				
FIB-4	0.95 ± 0.59	0.95 ± 0.60	0.93 ± 0.58	NS
FAST	0.10 ± 0.13	0.13 ± 0.15	0.06 ± 0.08	<0.001
FI	3.63 ± 1.73	3.86 ± 1.62	3.31 ± 1.82	<0.001
**Medications**				
Metformin (%)	278 (26.8)	198 (33.2)	80 (18.1)	<0.001
Anti-hypertensive drugs (%)	497 (47.8)	322 (53.9)	175 (39.6)	<0.001
Statin (%)	418 (40.2)	245 (41.0)	173 (39.1)	NS
**Medical history**				
Obesity (%)	504 (48.5)	397 (66.5)	107 (24.2)	<0.001
Hypertension (%)	599 (57.7)	393 (65.8)	206 (46.6)	<0.001
Diabetes (%)	411 (39.6)	301 (50.4)	110 (24.9)	<0.001
Insulin resistance (%)	591 (56.9)	430 (72.0)	161 (36.4)	<0.001
Dyslipidemia (%)	837 (80.6)	513 (85.9)	324 (73.3)	<0.001
MetS (IDF criteria) (%)	562 (54.1)	443 (74.2)	119 (26.9)	<0.001
MACE (%)	126 (12.1)	75 (12.6)	51 (11.5)	NS

Data are presented as mean±standard deviation and frequencies (%); P < 0.05 considered as statistically significant. **Abbreviations**: ALT, alanine transaminase; AST, aspartate transaminase; BMI, body mass index; CAP, controlled attenuation parameter; CHO, carbohydrates; FAST, FibroScan-AST score; FBG, fasting blood glucose; FIB-4, fibrosis-4 index; FI, fibrosis index; GGT; Gamma-glutamyl transferase, HDL, high-density lipoprotein; HOMA-IR, homeostatic model assessment of insulin resistance; IDF, International Diabetes Federation; LDL, low-density lipoprotein; LSM, liver stiffness measurement; MACE; Major adverse cardiovascular event, MetS, metabolic syndrome; SSM, spleen stiffness measurement; TAG, triglycerides; TC, total cholesterol.

### 3.2. Prevalence of MASLD and associated factors

**Fig 2:**
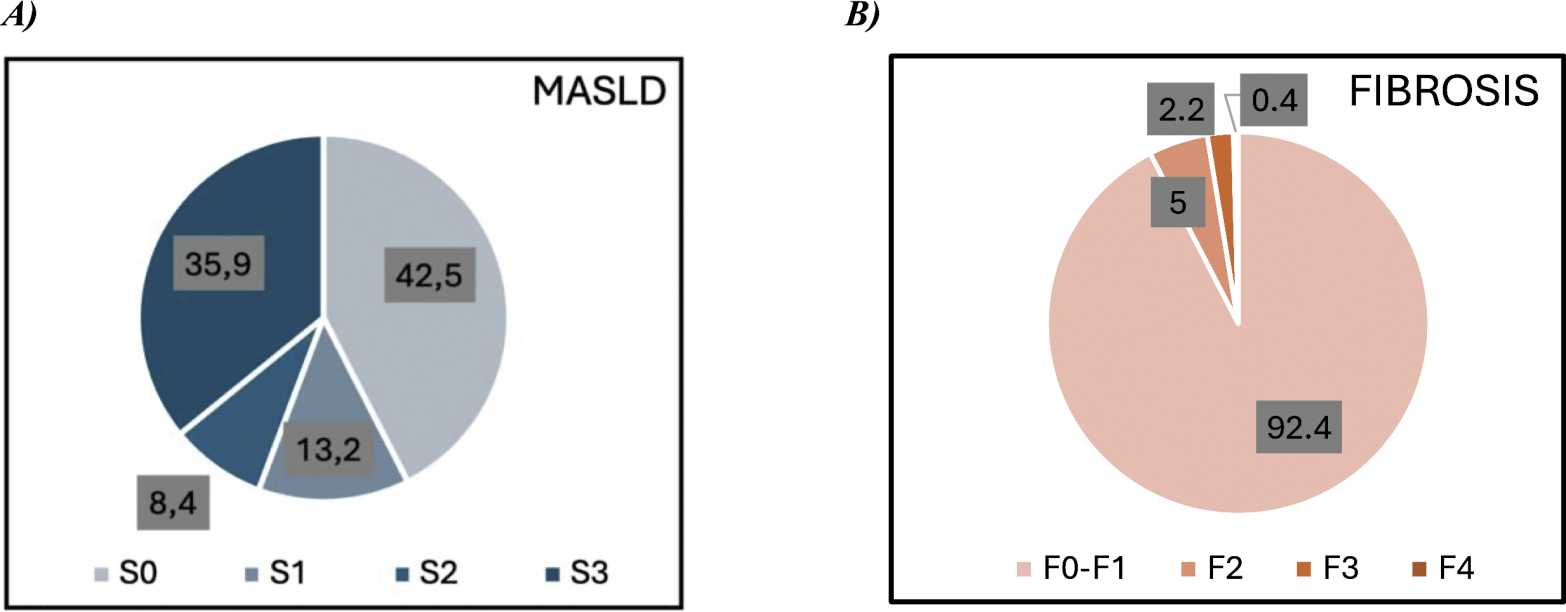
Stages of MASLD and fibrosis in the study population.

**Fig 3:**
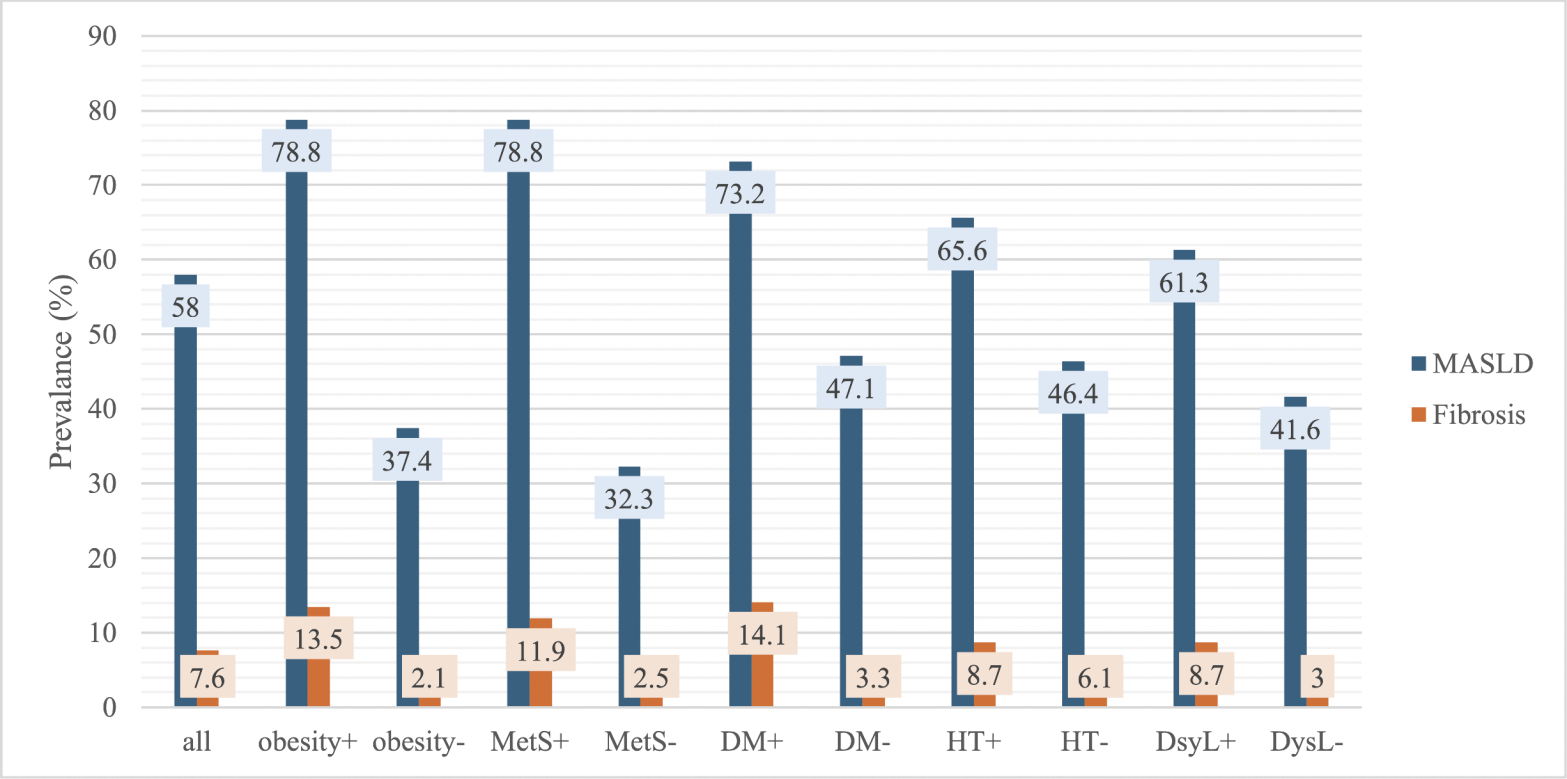
Prevalence of MASLD and significant fibrosis according to presence of obesity, metabolic syndrome, diabetes and hypertension. Abbreviations: DM, diabetes mellitus; DysL, dyslipidemia, HT, hypertension; MetS, metabolic syndrome.

The overall prevalence of MASLD, as determined by CAP, was 57.5% (95% CI: 54.4%–60.4%), with severe steatosis (S3) observed in 35.9% (95% CI: 33.0%–38.9%) of cases (Fig 2). When stratified by sex, males (59.8%, 95% CI: 55.5%–64.0%) had higher MASLD prevalence compared with females (55.1%95% CI: 50.9%–59.3%). MASLD was most prevalent among individuals with obesity and MetS, and least common in those without MetS. The largest disparity in MASLD prevalence was also observed between participants with and without MetS (Fig 3).

Subjects with MASLD were older, had lower educational attainment, and exhibited higher BMI and heart rate values. In terms of laboratory findings, MASLD cases showed significantly elevated levels of HbA1c, fasting blood glucose, ALT, AST, GGT, triglycerides, and HOMA-IR, along with significantly reduced HDL levels. Prevalence of obesity, MetS and all its components were higher in the MASLD group (Table 1). According to a multivariable logistic regression, independent factors associated with higher odds of MASLD were male sex, the presence of obesity, insulin resistance, MetS and central obesity, as well as the research site (Table 2, S4 Table). All necessary assumptions were met for the analysis in Table 2.

**Table 2:** Multivariable logistic regression analyses of factors associated with the presence of MASLD based on transient elastography.

	B	OR	95% CI for OR		p
**Research site**	**.976**	**2.653**	**1.862**	**3.781**	**<.001**
**Female sex**	**−.601**	**.548**	**.391**	**.770**	**<.001**
Age category					.195
18–34 (Ref.)					
35-49	.491	1.634	.954	2.801	.074
50-64	.595	1.813	1.055	3.114	.031
65-80	.512	1.668	.882	3.156	.116
**Obesity**	**.792**	**2.208**	**1.535**	**3.177**	**<.001**
Diabetes	.304	1.355	.951	1.931	.092
**Insulin resistance**	**.741**	**2.098**	**1.528**	**2.881**	**<.001**
Hypertension	.358	1.430	.992	2.061	.055
**Metabolic syndrome**	**.891**	**2.436**	**1.614**	**3.679**	**<.001**
**Central obesity**	**1.035**	**2.816**	**1.725**	**4.595**	**<.001**
**Constant**	**−3.589**	**.028**			**<.001**

a. Variable(s) entered on step 1: marital status, income level, education (years), alcohol, smoker, obesity, diabetes, insulin resistance, hypertension, dyslipidemia, metabolic syndrome, central obesity

### 3.3. Prevalence of fibrosis and associated factors

The overall prevalence of significant liver fibrosis, as determined by LSM, was 7.6% (95% CI: 6.1%–9.4%), with advanced fibrosis (>F3) observed in 2.6% (95% CI: 1.8%–3.8%) of cases (Fig 2). Among individuals with MASLD, the prevalence of significant fibrosis increased to 11.1% (95% CI: 8.8%–13.8%). When stratified by sex, females (8.2%, 95% CI: 6.1%–10.8%) had slightly higher fibrosis prevalence compared with males (7.0%, 95% CI: 5.1%–9.6%). Fibrosis was most prevalent among individuals with DM and obesity, and least common among those without obesity. The largest disparity in fibrosis prevalence was also observed between obese and non-obese participants (Fig 3).

Subjects with significant fibrosis had lower income, lower educational attainment and exhibited higher BMI and heart rate values. In terms of laboratory findings, fibrosis cases showed significantly elevated levels of HbA1c, fasting blood glucose, ALT, AST, GGT, LDL, triglycerides, and HOMA-IR, along with significantly reduced HDL levels. Prevalence of obesity, MetS and all its components were higher in the fibrosis group, whereas noninvasive indices of fibrosis were significantly lower (Table 3). On multivariable logistic regression, independent factors associated with higher odds of significant fibrosis were the presence of obesity, diabetes, MetS and insulin resistance, while hypertension was associated with lower odds of significant fibrosis. Compared to the very low-income group, the high-income group has a 75% reduction in the odds of significant fibrosis, when adjusting for research site, sex, age, marital status, obesity, diabetes, insulin resistance, hypertension, and metabolic syndrome (Table 4, S5 Table). All necessary assumptions were met for the analysis in Table 4.

**Table 3:** Characteristics of patients with and without advanced fibrosis based on transient elastography.

	All (n = 1039)	Fibrosis(n = 79)	No-Fibrosis (n = 960)	p
**Demographics**				
Age (years)	51.7 ± 13.1	53.4 ± 13.8	51.5 ± 13.0	NS
Male (%)	513 (49.4)	49 (45.8)	462 (49.8)	NS
Education (years)	12.1 ± 4.7	10.5 ± 5.3	12.3 ± 4.6	<0.001
*• Primary school (%)*	193 (18.6)	36 (33.6)	157 (16.8)	<0.001
*• Secondary school (%)*	94 (9.0)	7 (6.5)	87 (9.3)	<0.001
*• High School (%)*	285 (27.4)	28 (26.2)	257 (27.6)	NS
*• University or higher (%)*	467 (45.0)	36 (33.7)	431 (46.3)	<0.001
Married (%)	761 (73.2)	78 (72.2)	683 (73.3)	NS
Very low & Low Income (%)	147 (14.0)	15 (14.0)	132 (14.1)	NS
Medium & Medium High Income (%)	565 (54.4)	73 (68.2)	492 (52.7)	0.009
High Income (%)	327 (31.5)	19 (17.8)	308 (33.0)	0.008
**Anthropometrics**				
Weight (kg)	86.0 ± 17.5	98.6 ± 18.9	84.6 ± 16.7	<0.001
BMI (kg/m^2^)	30.5 ± 5.9	35.8 ± 6.0	29.9 ± 5.5	<0.001
Central obesity (%)	780 (75.1)	101 (94.4)	679 (72.9)	<0.001
**Lifestyle**				
Drinking (%)	410 (39.5)	28 (26.2)	382 (41.0)	0.003
Alcohol consumption (g/week)	43.6 ± 79.1	31.0 ± 73.1	45.1 ± 79.7	NS
Smoking (%)	304 (29.3)	25 (23.4)	279 (29.9)	NS
**Biochemical and Imaging Assessments**				
CAP (db/m)	260.1 ± 54.4	305.0 ± 47.9	254.9 ± 52.7	<0.001
LSM (kPa)	5.5 ± 2.1	9.7 ± 3.4	5.0 ± 1.2	<0.001
SSM (kPa) (n = 551)	26.4 ± 12.0	39.3 ± 20.6	25.5 ± 10.7	<0.001
HR (bpm)	70.8 ± 9.7	72.5 ± 10.1	70.6 ± 9.7	0.031
HgA1c (%) (n = 987)	5.9 ± 1.2	6.8 ± 1.5	5.8 ± 1.1	<0.001
FBG (mg/dL)	101.3 ± 38.7	123.0 ± 49.8	98.8 ± 36.4	<0.001
HOMA-IR	3.9 ± 4.0	7.0 ± 5.4	3.5 ± 3.7	<0.001
ALT (IU/L)	24.2 ± 17.4	33.1 ± 23.7	23.2 ± 16.2	<0.001
AST (IU/L)	21.0 ± 12.0	28.5 ± 21.6	20.1 ± 10.1	<0.001
GGT (IU/L)	28.4 ± 31.2	43.7 ± 42.9	26.6 ± 29.1	<0.001
TC (mg/dL)	196.0 ± 47.5	189.0 ± 45.8	196.8 ± 47.6	NS
LDL (mg/dL)	116.6 ± 41.6	108.9 ± 41.7	117.4 ± 41.7	0.048
HDL (mg/dL)	48.4 ± 14.5	43.3 ± 13.1	49.0 ± 14.6	<0.001
TAG (mg/dL)	153.6 ± 101.9	191.0 ± 105.4	149.4 ± 100.6	<0.001
Ferritin	125.3 ± 150.5	103.8 ± 93.5	127.6 ± 155.3	0.05
**Non-invasive indices**				
FIB-4	0.95 ± 0.59	1.24 ± 1.06	0.91 ± 0.50	<0.001
FAST	0.10 ± 0.13	0.27 ± 0.23	0.08 ± 0.1	<0.001
FI	3.63 ± 1.73	4.38 ± 2.01	3.54 ± 1.68	<0.001
**Medications**				
Metformin (%)	278 (26.8)	47 (43.9)	231 (24.8)	<0.001
Anti-hypertensive drugs (%)	497 (47.8)	62 (57.9)	435 (46.7)	0.027
Statin (%)	418 (40.2)	47 (43.9)	371 (39.8)	NS
**Medical history**				
Obesity (%)	504 (48.5)	94 (87.9)	410 (44.0)	<0.001
Hypertension (%)	599 (57.7)	66 (61.7)	533 (57.2)	NS
Diabetes (%)	411 (39.6)	73 (68.2)	338 (36.3)	<0.001
Insulin resistance (%)	591 (56.9)	96 (89.7)	495 (53.1)	<0.001
Dyslipidemia (%)	837 (80.6)	97 (90.7)	740 (79.4)	0.005
MetS (IDF criteria) (%)	562 (54.1)	91 (85)	471 (50.5)	<0.001
MACE (%)	126 (12.1)	21 (19.6)	105 (11.3)	0.012

Data are presented as mean±standard deviation and frequencies (%); P < 0.05 considered as statistically significant. **Abbreviations**: ALT, alanine transaminase; AST, aspartate transaminase; BMI, body mass index; CAP, controlled attenuation parameter; CHO, carbohydrates; FAST, FibroScan-AST score; FBG, fasting blood glucose; FIB-4, fibrosis-4 index; FI, fibrosis index; GGT; Gamma-glutamyl transferase, HDL, high-density lipoprotein; HOMA-IR, homeostatic model assessment of insulin resistance; IDF, International Diabetes Federation; LDL, low-density lipoprotein; LSM, liver stiffness measurement; MACE; Major adverse cardiovascular event, MetS, metabolic syndrome; SSM, spleen stiffness measurement; TAG, triglycerides; TC, total cholesterol. Values are presented as mean±SD.

**Table 4:** Multivariable logistic regression analyses of factors associated with the presence of significant fibrosis based on transient elastography.

	B	OR	95% CI for OR		p
Research site	.102	1.107	.667	1.836	.694
Female sex	−.239	.788	.483	1.283	.338
Age Category					.442
18–34 (Ref.)					
35-49	−.124	.884	.369	2.118	.782
50-64	.233	1.263	.533	2.994	.596
65-80	.447	1.563	.594	4.116	.366
**Income level**					**.049**
Very low (Ref.)					
Low	−1.024	.359	.091	1.415	.143
Middle	−.549	.577	.164	2.028	.392
Middle high	−.616	.540	.150	1.949	.347
**High**	**−1.361**	**.256**	**.069**	**.948**	**.041**
**Obesity**	**1.578**	**4.845**	**2.540**	**9.242**	**<.001**
**Diabetes**	**.775**	**2.172**	**1.306**	**3.610**	**.003**
**Insulin resistance**	**1.436**	**4.205**	**2.144**	**8.246**	**<.001**
**Hypertension**	**−.625**	**.535**	**.310**	**.923**	**.025**
**Metabolic Syndrome**	**.720**	**2.053**	**1.042**	**4.045**	**.038**
**Constant**	**−4.263**	**.014**			**<.001**

a. Variable(s) entered on step 1: marital status, income level, education (years), alcohol, smoker, obesity, diabetes, insulin resistance, hypertension, dyslipidemia, metabolic syndrome, central obesity

### 3.4. Sensitivity analysis

As sensitivity analysis, variable selections for the two multivariable logistic regression in Tables 2 and 4 were repeated without adjusting for insulin resistance. The overall pattern and magnitude of associations with MASLD and significant fibrosis remained consistent with the primary analyses. Diabetes maintained a modest but statistically significant association with MASLD (OR=1.43, 95% CI 1.01–2.02, p = 0.046). Key metabolic risk factors including obesity (OR=2.42, 95% CI 1.69–3.46, p < 0.001), metabolic syndrome (OR= 2.75, 95% CI 1.84–4.12, p < 0.001), high waist circumference (OR=2.93, 95% CI 1.81–4.74, p < 0.001), and hypertension (OR 1.47, 95% CI 1.03–2.11, p = 0.036) remained significant predictors of MASLD. Female sex continued to be inversely associated with MASLD (OR= 0.51, 95% CI 0.36–0.71, p < 0.001). Likewise for significant fibrosis, diabetes remained significantly associated, with an effect size nearly identical to the primary analysis (OR= 2.2, 95% CI 1.34–3.67, p = 0.002). Obesity continued to be the strongest metabolic correlate of significant fibrosis (OR= 5.65, 95% CI 2.97–10.72, p < 0.001), and metabolic syndrome retained a significant association (OR= 2.63, 95% CI 1.35–5.10, p = 0.004). Full results are presented in S6 and S7 Tables.

## 4. Discussion

This multicenter cross-sectional study conducted among individuals at risk for MASLD in Turkey demonstrated that more than half of those with at least one metabolic risk factor were affected by MASLD. In contrast, the prevalence of liver fibrosis in this population was considerably lower. To the best of our knowledge, this is the first study to date utilizing VCTE to assess MASLD and fibrosis in metabolically at-risk Turkish populations.

Our findings indicate a lower MASLD prevalence compared to some earlier VCTE-based screening studies in at-risk populations, which have reported prevalence rates ranging from 61.3% to 83.6% [25–30]. This discrepancy may, in part, be attributed to differences in inclusion criteria across studies. While only a few smaller studies included individuals with at least one metabolic risk factor—similar to our approach [31–33], the majority of investigations targeting at-risk MASLD populations have focused specifically on individuals with diabetes and/or obesity—conditions already known to be strongly associated with higher MASLD prevalence. Indeed, meta-analyses have reported MASLD rates of 69.9% in overweight individuals, 75.3% in obese individuals and 65.3% in those with diabetes [34,35]. A recent meta-analysis focusing on studies from the Middle East reported a MASLD prevalence of 68.7% in the region and 70.0% in Turkey among patients with type 2 diabetes [3]. In contrast, MASLD appears to be less common in individuals with hypertension or dyslipidemia, with lower reported rates among these subgroups [36]. Consistent with this pattern, our study also found substantially higher MASLD prevalence in obese and diabetic participants compared to those with hypertension or dyslipidemia. These findings align with previous sporadic reports on MASLD prevalence among Turkish patients with diabetes [12–14] and obesity [15]. Although earlier Turkish reports, despite their methodological limitations, yielded MASLD prevalence estimates consistent with international data, our study provides a more robust confirmation of them, strengthening the evidence for MASLD burden in Turkish at-risk populations.

In our study, male sex was associated with an increased odds of MASLD, in line with previous evidence [11,32,37–41]. Among clinical predictors, central obesity emerged as the strongest determinant of MASLD, followed by general obesity. Although obesity defined by BMI has consistently been associated with MASLD [4,7,11,14,42], central obesity had not been previously investigated in Turkey. Worldwide evidence suggests that fat distribution, rather than overall fat mass, may play a more critical role in the pathogenesis of MASLD [43,44]. While it remains unclear whether the effect of central obesity is independent of general obesity, our findings address this gap and suggest that central adiposity may exert a comparable—if not greater—impact on MASLD risk in Turkish populations. This is similar to the findings from Asian populations, where both central and general obesity have been shown to similarly elevate MASLD risk [45]. Furthermore, dysfunctional adipose tissue in central obesity also causes insulin resistance – a key pathophysiological mechanism responsible for the development and progression of MASLD. Insulin resistance promotes adipose tissue lipolysis, leading to elevated circulating free fatty acids and their subsequent deposition in the liver, resulting in hepatic steatosis [46]. In line with this, we showed that the presence of insulin resistance, but not diabetes, is an independent predictor of MASLD in Turkey, which corroborates previous studies linking insulin resistance markers to increased MASLD risk [47,48]. The lack of a significant association between diabetes and MASLD in our study contrasts with many population-based reports but likely reflects the specific characteristics of our at-risk cohort. Because all participants fulfilled at least one metabolic criterion for MASLD diagnosis, the background prevalence of metabolic disturbances was already high, diminishing the discriminatory effect of diabetes on steatosis presence. Moreover, most individuals with diabetes had relatively well-controlled glycemia (mean HbA1c = 6.6 ± 1.5) and were under active treatment, which may have limited hepatic fat accumulation. The presence of MetS, on the other hand, showed the second strongest association with MASLD in our cohort, corresponding to a 2.5-fold increase in the odds of disease presence. In comparison, a cohort study involving 11,647 individuals reported an 11.5-fold increase for the same association [49]. However, their reliance on ultrasound for MASLD diagnosis may have compromised the accuracy of the findings. Notably, studies employing VCTE as the diagnostic modality have reported results more consistent with ours [18,31,32]. Finally, subjects enrolled at the tertiary center showed considerably higher odds of MASLD than those from the outpatient clinic, which may be explained by several factors. These include referral bias, as tertiary centers often manage more complex cases; and a higher burden of underlying comorbidities that we did not adjust for. Additionally, patients at tertiary centers may exhibit distinct health-seeking behaviors, potentially reflecting more advanced or symptomatic disease. Taken together, these findings reiterate the need for further research to better characterize MASLD risk factors in at-risk populations in various clinical settings.

To date, only a limited number of studies have investigated the prevalence of liver fibrosis in at-risk populations. Three nationwide studies and a large cross-sectional study consistently reported similar prevalence rates, ranging between 13–14% in individuals with metabolic syndrome, 11–14% in those with obesity, %12 in central obesity and 14–21% in patients with diabetes [50–53]. Two relatively small studies that included individuals with at least one metabolic risk factor—similar to our inclusion criteria—reported fibrosis prevalences of 9.4% and 16.8%, respectively [31,32]. In line with these findings, our results demonstrated that fibrosis was most prevalent among participants with obesity, diabetes, and MetS, with subgroup-specific rates comparable to those reported in previous studies. However, earlier studies conducted in Turkish cohorts have yielded different prevalence estimates. For example, fibrosis was reported in 6.6% and 9.8% of patients presenting to tertiary centers for dyspepsia [5,54]. Among adults with diabetes, prevalence ranged from 9.5% to 16.9% [12–14], while a study in apparently healthy individuals reported a rate of 15.8% [7]. These discrepancies likely reflect differences in study populations, settings, and possibly methodological approaches, suggesting that previous cohorts may not be representative of the broader at-risk population in Turkey. By focusing on a well-defined at-risk population using standardized assessment methods, our study helps address this gap and provides prevalence estimates that are consistent with international figures.

In our study, obesity emerged as the strongest predictor of significant liver fibrosis, consistent with previous reports demonstrating a similar association between obesity and fibrosis risk [50,55–57]. This relationship may be interpreted in several ways: obesity may directly contribute to fibrogenesis, or alternatively, it may exert its effect indirectly by promoting metabolic disturbances such as impaired glucose metabolism, which are known contributors to liver fibrosis. Supporting this, our findings -as well as those of other studies [52,58–60]- highlight insulin resistance and diabetes as strong predictors of clinically significant fibrosis. These observations underscore the critical role of these potentially modifiable risk factors in fibrosis progression and emphasize the importance of early intervention targeting insulin resistance and/or diabetes. Adjusted for its individual components, MetS itself was independently associated with fibrosis in our cohort. Very few studies have examined the role of MetS in hepatic fibrosis. One study reported more than a three-fold increase in fibrosis risk among individuals with MetS compared to those without [53], whereas a biopsy-proven MASLD cohort from Turkey did not identify MetS as a significant predictor of fibrosis [61]. These conflicting findings highlight the need for larger, prospective studies to clarify the independent contribution of MetS to hepatic fibrosis risk. We also observed an unexpected inverse association between hypertension and significant fibrosis. While this finding aligns with results from prior work reporting an attenuated risk among hypertensive individuals [62], it should be interpreted cautiously, as unmeasured confounding, treatment effects, or selection bias may partially explain this association. Finally, we observed a significantly lower odds of fibrosis among individuals in the highest income group. A similar trend was reported in a nationwide study involving 5.7 million participants, where fibrosis prevalence gradually declined with increasing GDP per capita, although the associations did not reach statistical significance [50]. This may reflect the complex and context-dependent relationship between socioeconomic status and liver health, which can be influenced by various mediating factors such as healthcare access, lifestyle habits, and comorbidities. Further research is warranted to explore these pathways and determine whether income-related disparities contribute to fibrosis risk in different populations.

The strengths of the present study include a relatively large sample, and the use of a reliable, imaging-based, non-invasive technique to assess hepatic steatosis and fibrosis, offering advantages over surrogate blood-based markers. Although MASLD has been widely studied in different regions, our study fills a major knowledge gap by providing the first multicenter, VCTE-based assessment of steatosis and fibrosis prevalence among metabolically at-risk adults in Turkey—a population with one of the world’s highest rates of obesity and type 2 diabetes. The standardized design across centers and exclusion of secondary liver disease sources strengthen the reliability and comparability of our findings. Importantly, the identification of fibrosis-related metabolic phenotypes within this high-risk cohort highlights specific subgroups who may benefit from early MASLD screening in metabolic or endocrine clinics. These results carry direct clinical implications for prioritizing non-invasive liver assessment in cardiometabolic care pathways and for informing national strategies aimed at early detection and prevention of advanced liver disease. Moreover, although this was not a nationwide study, the socioeconomic characteristics of the study population were moderately representative of the broader population, thereby enhancing the generalizability of our findings. Nonetheless, as the study was partially conducted at a tertiary care center and included individuals with established risk factors, the metabolic burden among participants was likely higher than that of the general population. Therefore, the findings may not be generalizable to the ostensibly healthy Turkish population. Our findings should be interpreted also in light of several limitations. Although we identified important associations, the observational nature of the study precludes conclusions regarding causality. Additionally, we did not use the gold standard of liver biopsy to assess fibrosis. However, performing liver biopsies in all participants of a cohort would have been impractical, and VCTE remains a validated and widely accepted non-invasive tool for fibrosis assessment. Although the inclusion of both an outpatient clinic and a tertiary care center enhanced the diversity and size of our sample, it may have introduced selection bias. Patients attending tertiary centers are typically referred for more complex or advanced health conditions and may differ systematically from those seen in primary or outpatient settings. As such, the higher prevalence of MASLD observed among participants from the tertiary center may reflect an overrepresentation of individuals with more severe metabolic risk profiles. Also, study site was included as a fixed effect to account for systematic differences between centers; however, this approach does not explicitly model within-site correlation, which should be considered when interpreting the results. Finally, as the study was conducted in a metropolitan setting, our findings may not fully reflect the epidemiological patterns of MASLD in rural populations in Turkey.

To conclude, this study demonstrated that the prevalence of MASLD in at-risk populations in Turkey is considerably high, while MASLD-related signficant fibrosis remains less common. Obesity, insulin resistance, and metabolic syndrome emerged as shared risk factors for both conditions. Our findings support targeted screening and monitoring of these high-risk groups to identify individuals at greater risk for advanced MASLD. Nonetheless, further research is needed to evaluate the long-term benefits and cost-effectiveness of such an approach.

## Supporting information

S1 Table Inclusion and exclusion criteria. (DOCX)

S2 Table Comparison of the socio-economic factors with Turkish national averages. (DOCX)

S3 Table Baseline characteristics of research sites. (DOCX)

S4 Table Multivariable logistic regression analyses of factors associated with the presence of MASLD based on transient elastography. (DOCX)

S5 Table Multivariable logistic regression analyses of factors associated with the presence of significant fibrosis based on transient elastography. (DOCX)

S6 Table Sensitivity analyses without insulin resistance for factors associated with the presence of MASLD based on transient elastography. (DOCX)

S7 Table Sensitivity analyses without insulin resistance for factors associated with the presence of significant fibrosis based on transient elastography. (DOCX)
