# Supplementary material for: Prevalence of MASLD and fibrosis in Turkey: Results from a multicenter study of at-risk populations
Source: PLoS One. 2026 Feb 12;21(2):e0341214. doi: 10.1371/journal.pone.0341214 (PMC12900293; doi:10.1371/journal.pone.0341214)
Supplement: S1 Table — (DOCX) [file pone.0341214.s001.docx]

**S1 Table. Inclusion and exclusion criteria**

| **Inclusion Criteria:**  Able to understand and sign the informed consent  Able to speak Turkish  Between 18-80 years  Having one of the following conditions:   - BMI >25 kg/m² or waist circumference ≥94 cm in men, ≥80 cm in women - Insulin resistance/Impaired glucose tolerence: HbA1c 5.7-6.4% or fasting plasma glucose 100-125 mg/dl - Type 2 diabetes mellitus: HbA1c ≥6.5% or fasting plasma glucose ≥126 mg/dl - Treatment for type 2 diabetes - Hypertension: ≥130/85 mmHg or treatment for hypertension - Dyslipidemia: Triglicerides ≥150 mg/dl or HDL ≤39 mg/dl in men, ≤50 mg/dl in women or lipid-lowering treatment. | **Exclusion Criteria:**  Excessive alcohol use (more than 20 g/day for women and 30g/day for men= >2 glasses alcohol/day for women and >3 glasses for men)  Other liver diseases: Hepatitis B virus, Hepatitis C virus, autoimmune hepatitis, primary biliary cirrhosis, hemochromatosis, Wilson's disease, Alpha 1 antitrypsin deficiency  Secondary causes for steatosis: disorders of lipid metabolism, HCV Genotype 3, total parental nutrition, severe surgical weight loss, medications (amiodarone, tamoxifen, methotrexate, corticosteroids and HAART), lean steatosis, Celiac disease, environmental toxicity  Pregnancy and breastfeeding.  A history of bariatric surgery.  Diagnosis of liver cirrhosis and/or hepatocellular carcinoma.  Current diagnosis of extrahepatic malignancy(s) or prior diagnosis within last 5 years. |
| --- | --- |
