# Supplementary material for: Prevalence of MASLD and fibrosis in Turkey: Results from a multicenter study of at-risk populations
Source: PLoS One. 2026 Feb 12;21(2):e0341214. doi: 10.1371/journal.pone.0341214 (PMC12900293; doi:10.1371/journal.pone.0341214)
Supplement: S2 Table — (DOCX) [file pone.0341214.s002.docx]

**S2 Table. Comparison of the socio-economic factors with Turkish national averages**

|  | **National** | **Overall Cohort** | **Site 1: Outpatient** | **Site 2: Tertiary care** |
| --- | --- | --- | --- | --- |
| **Age Category (%)** | | | | |
| 18-34 | 25.3 | 11.1 | 11.2 | 10.9 |
| 35-49 | 21.9 | 29.7 | 30.5 | 28.8 |
| 50-64 | 16.7 | 43.8 | 42.4 | 45.5 |
| 65-80 | 10.6 | 15.4 | 15.9 | 14.8 |
| **Education (%)** | | | | |
| primary | 31.2 | 18.6 | 6.7 | 33.6 |
| secondary | 13.0 | 9.0 | 6.9 | 11.8 |
| high school | 23.5 | 27.4 | 27.2 | 27.7 |
| university | 21.0 | 36.5 | 46.4 | 24.0 |
| postgraduate | 3.4 | 8.5 | 12.8 | 3.1 |
| **Marital Status (%)** | | | | |
| single | 28.8 | 16.3 | 17.9 | 14.2 |
| married | 60.6 | 73.2 | 70.0 | 77.3 |
| divorced | 10.6 | 10.5 | 12.1 | 8.5 |
| **Income (%)** | | | | |
| very low | 6.3 | 2.0 | 2.6 | 1.3 |
| low | 10.4 | 12.1 | 11.6 | 12.9 |
| middle | 14.6 | 31.1 | 27.6 | 35.5 |
| middle high | 20.7 | 23.3 | 20.5 | 26.8 |
| high | 48.1 | 31.5 | 37.8 | 23.5 |
