# Supplementary material for: Prevalence of MASLD and fibrosis in Turkey: Results from a multicenter study of at-risk populations
Source: PLoS One. 2026 Feb 12;21(2):e0341214. doi: 10.1371/journal.pone.0341214 (PMC12900293; doi:10.1371/journal.pone.0341214)
Supplement: S3 Table — (DOCX) [file pone.0341214.s003.docx]

**S3 Table. Baseline characteristics of research sites**

|  | **Site 1: outpatient clinic** | **Site 2: tertiary care center** |
| --- | --- | --- |
| **Age (years)** | 51.8 ±13.2 | 51.7±12.7 |
| **Education (years)** | 13.8±3.8 | 10.0±4.9 |
| **Male (%)** | 363 (62.6) | 150 (32.7) |
| **Weight (kg)** | 83.7±16.5 | 89.0±18.2 |
| **Body mass index (kg/m2)** | 28.4±4.4 | 33.3±6.3 |
| **Heart rate (bpm)** | 71.2±11.0 | 70.2±7.8 |
| **CAP (db/m)** | 243.9±50.0 | 280.5±52.7 |
| **LSM (kPa)** | 5.2±2.2 | 5.7±2.0 |
| **ALT (IU/L)** | 26.9±18.0 | 20.9±16.0 |
| **AST (IU/L)** | 22.9±13.5 | 18.5±9.4 |
| **GGT (IU/L)** | 29.6±32.5 | 26.8±29.5 |
| **Fasting glucose (mg/dL)** | 95.9±29.0 | 108.2±47.3 |
| **HbA1c (%)** | 5.7±1.0 | 6.2±1.4 |
| **HOMA_IR** | 3.4±3.9 | 4.5±4.2 |
| **Total cholesterol (mg/dL)** | 197.1±51.9 | 194.7±41.3 |
| **HDL cholesterol (mg/dL)** | 50.0±14.4 | 46.5±14.5 |
| **LDL cholesterol (mg/dL)** | 119.0±44.5 | 113.4±37.4 |
| **Triglycerides (mg/dL)** | 147.4±96.4 | 161.5±107.9 |
| **FIB4** | 1.0±0.6 | 0.9±0.51 |
| **FAST** | 0.1±0.1 | 0.1±0.1 |
| **MASLD (%)** | 268 (46.2) | 329 (71.7) |
| **Significant fibrosis (%)** | 38 (6.6) | 69 (5.0) |
| **Obesity (%)** | 196 (33.8) | 308 (67.1) |
| **Type 2 diabetes(%)** | 173 (29.8) | 238 (51.9) |
| **Insulin resistance(%)** | 282 (48.6) | 309 (67.3) |
| **Hypertension (%)** | 367 (63.3) | 232 (50.5) |
| **Dyslipidemia(%)** | 463 (79.8) | 374 (81.5) |
| **Metabolic syndrome%)** | 289 (49.8) | 273 (83.2) |
| **Central obesity (%)** | 398 (68.6) | 382 (83.2) |

Data are presented as mean±standard deviation and frequencies (%)

**Abbreviations**: ALT, alanine transaminase; AST, aspartate transaminase; CAP, controlled attenuation parameter; FAST, FibroScan-AST score; FIB-4, fibrosis-4 index; FI, fibrosis index; GGT; Gamma-glutamyl transferase, HDL, high-density lipoprotein; HOMA-IR, homeostatic model assessment of insulin resistance; IDF, International Diabetes Federation; LDL, low-density lipoprotein; LSM, liver stiffness measurement;TAG, triglycerides; TC, total cholesterol.
