# Supplementary material for: Prevalence of MASLD and fibrosis in Turkey: Results from a multicenter study of at-risk populations
Source: PLoS One. 2026 Feb 12;21(2):e0341214. doi: 10.1371/journal.pone.0341214 (PMC12900293; doi:10.1371/journal.pone.0341214)
Supplement: S4 Table — (DOCX) [file pone.0341214.s004.docx]

**S4 Table. Multivariable logistic regression analyses of factors associated with the presence of MASLD based on transient elastography**

|  | | B | S.E. | Wald | df | Sig. | Exp(B) | 95% C.I.for EXP(B) | |
| --- | --- | --- | --- | --- | --- | --- | --- | --- | --- |
|  |  |  |  |  |  |  |  | Lower | Upper |
| Step 1^a^ | Age category |  |  | 3.744 | 3 | .290 |  |  |  |
|  | age_cat(1) | .471 | .286 | 2.715 | 1 | .099 | 1.601 | .915 | 2.804 |
|  | age_cat(2) | .558 | .291 | 3.664 | 1 | .056 | 1.747 | .987 | 3.093 |
|  | age_cat(3) | .477 | .344 | 1.920 | 1 | .166 | 1.611 | .821 | 3.164 |
|  | site | 1.015 | .201 | 25.609 | 1 | <.001 | 2.759 | 1.862 | 4.087 |
|  | sex | -.562 | .184 | 9.295 | 1 | .002 | .570 | .397 | .818 |
|  | Education (years) | .000 | .020 | .000 | 1 | .984 | 1.000 | .961 | 1.039 |
|  | Marital status | -.036 | .158 | .053 | 1 | .817 | .964 | .708 | 1.313 |
|  | income |  |  | 6.806 | 4 | .147 |  |  |  |
|  | income(1) | 1.128 | .583 | 3.744 | 1 | .053 | 3.090 | .985 | 9.689 |
|  | income(2) | .757 | .556 | 1.856 | 1 | .173 | 2.132 | .717 | 6.338 |
|  | income(3) | .532 | .565 | .886 | 1 | .347 | 1.703 | .562 | 5.158 |
|  | income(4) | .839 | .561 | 2.233 | 1 | .135 | 2.313 | .770 | 6.947 |
|  | alcohol | .080 | .197 | .165 | 1 | .685 | 1.083 | .736 | 1.593 |
|  | smoker | -.005 | .090 | .003 | 1 | .955 | .995 | .833 | 1.188 |
|  | obesity | .815 | .188 | 18.726 | 1 | <.001 | 2.259 | 1.562 | 3.268 |
|  | DM | .309 | .183 | 2.854 | 1 | .091 | 1.362 | .952 | 1.950 |
|  | IR | .742 | .163 | 20.730 | 1 | <.001 | 2.100 | 1.526 | 2.890 |
|  | HT | .400 | .190 | 4.438 | 1 | .035 | 1.491 | 1.028 | 2.163 |
|  | Dyslipidemia | .275 | .216 | 1.623 | 1 | .203 | 1.316 | .862 | 2.009 |
|  | MetS | .825 | .223 | 13.641 | 1 | <.001 | 2.281 | 1.473 | 3.533 |
|  | highWC | 1.085 | .258 | 17.626 | 1 | <.001 | 2.959 | 1.783 | 4.909 |
|  | Constant | -4.642 | .843 | 30.297 | 1 | <.001 | .010 |  |  |
| Step 2^a^ | Age category |  |  | 3.808 | 3 | .283 |  |  |  |
|  | age_cat(1) | .471 | .285 | 2.744 | 1 | .098 | 1.602 | .917 | 2.799 |
|  | age_cat(2) | .559 | .289 | 3.729 | 1 | .053 | 1.748 | .992 | 3.082 |
|  | age_cat(3) | .478 | .342 | 1.947 | 1 | .163 | 1.612 | .824 | 3.154 |
|  | site | 1.016 | .191 | 28.227 | 1 | <.001 | 2.762 | 1.899 | 4.018 |
|  | sex | -.562 | .184 | 9.300 | 1 | .002 | .570 | .397 | .818 |
|  | Marital status | -.036 | .155 | .054 | 1 | .817 | .965 | .712 | 1.307 |
|  | Income level |  |  | 6.881 | 4 | .142 |  |  |  |
|  | income(1) | 1.129 | .582 | 3.755 | 1 | .053 | 3.092 | .987 | 9.682 |
|  | income(2) | .757 | .556 | 1.858 | 1 | .173 | 2.133 | .718 | 6.338 |
|  | income(3) | .532 | .565 | .886 | 1 | .347 | 1.702 | .562 | 5.153 |
|  | income(4) | .838 | .561 | 2.234 | 1 | .135 | 2.312 | .770 | 6.939 |
|  | alcohol | .080 | .196 | .164 | 1 | .685 | 1.083 | .737 | 1.592 |
|  | smoker | -.005 | .090 | .003 | 1 | .955 | .995 | .833 | 1.188 |
|  | obesity | .815 | .188 | 18.772 | 1 | <.001 | 2.260 | 1.563 | 3.267 |
|  | DM | .309 | .183 | 2.867 | 1 | .090 | 1.363 | .952 | 1.950 |
|  | IR | .742 | .163 | 20.729 | 1 | <.001 | 2.100 | 1.526 | 2.890 |
|  | HT | .400 | .190 | 4.451 | 1 | .035 | 1.492 | 1.029 | 2.163 |
|  | Dyslipidemia | .275 | .216 | 1.623 | 1 | .203 | 1.316 | .862 | 2.008 |
|  | MetS | .824 | .223 | 13.714 | 1 | <.001 | 2.280 | 1.474 | 3.527 |
|  | High WC | 1.085 | .257 | 17.856 | 1 | <.001 | 2.960 | 1.789 | 4.897 |
|  | Constant | -4.650 | .743 | 39.194 | 1 | <.001 | .010 |  |  |
| Step 3^a^ | Age category |  |  | 3.856 | 3 | .277 |  |  |  |
|  | age_cat(1) | .472 | .284 | 2.759 | 1 | .097 | 1.603 | .919 | 2.799 |
|  | age_cat(2) | .560 | .288 | 3.784 | 1 | .052 | 1.751 | .996 | 3.079 |
|  | age_cat(3) | .480 | .340 | 1.987 | 1 | .159 | 1.616 | .829 | 3.149 |
|  | site | 1.016 | .191 | 28.239 | 1 | <.001 | 2.762 | 1.899 | 4.018 |
|  | sex | -.562 | .184 | 9.300 | 1 | .002 | .570 | .397 | .818 |
|  | Marital status | -.036 | .155 | .055 | 1 | .815 | .964 | .712 | 1.306 |
|  | Income level |  |  | 6.886 | 4 | .142 |  |  |  |
|  | income(1) | 1.129 | .582 | 3.760 | 1 | .053 | 3.092 | .988 | 9.681 |
|  | income(2) | .758 | .555 | 1.861 | 1 | .173 | 2.134 | .718 | 6.338 |
|  | income(3) | .532 | .565 | .887 | 1 | .346 | 1.703 | .563 | 5.152 |
|  | income(4) | .838 | .561 | 2.237 | 1 | .135 | 2.313 | .771 | 6.938 |
|  | alcohol | .077 | .192 | .162 | 1 | .687 | 1.080 | .742 | 1.572 |
|  | obesity | .815 | .188 | 18.777 | 1 | <.001 | 2.259 | 1.562 | 3.265 |
|  | DM | .309 | .183 | 2.864 | 1 | .091 | 1.362 | .952 | 1.949 |
|  | IR | .742 | .163 | 20.728 | 1 | <.001 | 2.100 | 1.526 | 2.890 |
|  | HT | .400 | .189 | 4.458 | 1 | .035 | 1.492 | 1.029 | 2.163 |
|  | Dyslipidemia | .275 | .216 | 1.621 | 1 | .203 | 1.316 | .862 | 2.008 |
|  | MetS | .825 | .222 | 13.739 | 1 | <.001 | 2.281 | 1.475 | 3.527 |
|  | High WC | 1.086 | .257 | 17.870 | 1 | <.001 | 2.961 | 1.790 | 4.898 |
|  | Constant | -4.659 | .725 | 41.240 | 1 | <.001 | .009 |  |  |
| Step 4^a^ | Age category |  |  | 3.809 | 3 | .283 |  |  |  |
|  | age_cat(1) | .462 | .281 | 2.706 | 1 | .100 | 1.587 | .915 | 2.753 |
|  | age_cat(2) | .550 | .284 | 3.736 | 1 | .053 | 1.733 | .992 | 3.025 |
|  | age_cat(3) | .468 | .336 | 1.934 | 1 | .164 | 1.597 | .826 | 3.087 |
|  | site | 1.017 | .191 | 28.325 | 1 | <.001 | 2.765 | 1.901 | 4.022 |
|  | sex | -.561 | .184 | 9.276 | 1 | .002 | .571 | .398 | .819 |
|  | Income level |  |  | 6.887 | 4 | .142 |  |  |  |
|  | income(1) | 1.128 | .582 | 3.762 | 1 | .052 | 3.090 | .988 | 9.659 |
|  | income(2) | .757 | .555 | 1.863 | 1 | .172 | 2.132 | .719 | 6.324 |
|  | income(3) | .531 | .564 | .884 | 1 | .347 | 1.700 | .563 | 5.135 |
|  | income(4) | .836 | .560 | 2.233 | 1 | .135 | 2.308 | .771 | 6.912 |
|  | alcohol | .078 | .191 | .167 | 1 | .683 | 1.081 | .743 | 1.574 |
|  | obesity | .816 | .188 | 18.878 | 1 | <.001 | 2.262 | 1.565 | 3.270 |
|  | DM | .306 | .182 | 2.825 | 1 | .093 | 1.358 | .950 | 1.942 |
|  | IR | .740 | .163 | 20.673 | 1 | <.001 | 2.095 | 1.523 | 2.882 |
|  | HT | .398 | .189 | 4.428 | 1 | .035 | 1.490 | 1.028 | 2.159 |
|  | Dyslipidemia | .272 | .215 | 1.596 | 1 | .206 | 1.313 | .861 | 2.002 |
|  | MetS | .829 | .222 | 13.982 | 1 | <.001 | 2.291 | 1.484 | 3.537 |
|  | highWC | 1.083 | .257 | 17.818 | 1 | <.001 | 2.953 | 1.786 | 4.882 |
|  | Constant | -4.681 | .719 | 42.357 | 1 | <.001 | .009 |  |  |
| Step 5^a^ | Age category |  |  | 3.690 | 3 | .297 |  |  |  |
|  | age_cat(1) | .453 | .280 | 2.612 | 1 | .106 | 1.572 | .908 | 2.722 |
|  | age_cat(2) | .538 | .283 | 3.614 | 1 | .057 | 1.712 | .983 | 2.981 |
|  | age_cat(3) | .447 | .333 | 1.810 | 1 | .179 | 1.564 | .815 | 3.002 |
|  | site | .996 | .184 | 29.411 | 1 | <.001 | 2.706 | 1.888 | 3.878 |
|  | sex | -.581 | .178 | 10.621 | 1 | .001 | .560 | .395 | .793 |
|  | Income level |  |  | 6.849 | 4 | .144 |  |  |  |
|  | income(1) | 1.131 | .581 | 3.790 | 1 | .052 | 3.100 | .992 | 9.685 |
|  | income(2) | .768 | .554 | 1.922 | 1 | .166 | 2.155 | .728 | 6.381 |
|  | income(3) | .547 | .562 | .946 | 1 | .331 | 1.728 | .574 | 5.203 |
|  | income(4) | .858 | .557 | 2.375 | 1 | .123 | 2.359 | .792 | 7.027 |
|  | obesity | .808 | .187 | 18.736 | 1 | <.001 | 2.243 | 1.556 | 3.233 |
|  | DM | .305 | .182 | 2.811 | 1 | .094 | 1.357 | .950 | 1.940 |
|  | IR | .741 | .163 | 20.779 | 1 | <.001 | 2.099 | 1.526 | 2.887 |
|  | HT | .398 | .189 | 4.425 | 1 | .035 | 1.489 | 1.028 | 2.158 |
|  | Dyslipidemia | .269 | .215 | 1.556 | 1 | .212 | 1.308 | .858 | 1.994 |
|  | MetS | .824 | .221 | 13.866 | 1 | <.001 | 2.279 | 1.477 | 3.516 |
|  | High WC | 1.086 | .256 | 17.953 | 1 | <.001 | 2.964 | 1.793 | 4.899 |
|  | Constant | -4.607 | .695 | 43.890 | 1 | <.001 | .010 |  |  |
| Step 6^a^ | Age category |  |  | 4.595 | 3 | .204 |  |  |  |
|  | age_cat(1) | .493 | .277 | 3.168 | 1 | .075 | 1.638 | .951 | 2.818 |
|  | age_cat(2) | .592 | .279 | 4.522 | 1 | .033 | 1.808 | 1.047 | 3.121 |
|  | age_cat(3) | .516 | .327 | 2.486 | 1 | .115 | 1.676 | .882 | 3.184 |
|  | site | .999 | .183 | 29.655 | 1 | <.001 | 2.715 | 1.895 | 3.889 |
|  | Sex | -.603 | .177 | 11.598 | 1 | <.001 | .547 | .387 | .774 |
|  | Income level |  |  | 6.528 | 4 | .163 |  |  |  |
|  | income(1) | 1.095 | .580 | 3.564 | 1 | .059 | 2.989 | .959 | 9.317 |
|  | income(2) | .736 | .553 | 1.771 | 1 | .183 | 2.087 | .706 | 6.165 |
|  | income(3) | .526 | .562 | .877 | 1 | .349 | 1.692 | .563 | 5.090 |
|  | income(4) | .834 | .556 | 2.247 | 1 | .134 | 2.302 | .774 | 6.847 |
|  | obesity | .812 | .187 | 18.925 | 1 | <.001 | 2.252 | 1.562 | 3.247 |
|  | DM | .293 | .182 | 2.602 | 1 | .107 | 1.341 | .939 | 1.914 |
|  | IR | .747 | .163 | 21.110 | 1 | <.001 | 2.111 | 1.535 | 2.903 |
|  | HT | .372 | .188 | 3.935 | 1 | .047 | 1.451 | 1.004 | 2.097 |
|  | MetS | .904 | .212 | 18.241 | 1 | <.001 | 2.469 | 1.631 | 3.739 |
|  | High WC | 1.034 | .252 | 16.871 | 1 | <.001 | 2.812 | 1.717 | 4.605 |
|  | Constant | -4.388 | .669 | 42.998 | 1 | <.001 | .012 |  |  |
| Step 7^a^ | Age category |  |  | 4.699 | 3 | .195 |  |  |  |
|  | age_cat(1) | .491 | .275 | 3.193 | 1 | .074 | 1.634 | .954 | 2.801 |
|  | age_cat(2) | .595 | .276 | 4.642 | 1 | .031 | 1.813 | 1.055 | 3.114 |
|  | age_cat(3) | .512 | .325 | 2.475 | 1 | .116 | 1.668 | .882 | 3.156 |
|  | site | .976 | .181 | 29.140 | 1 | <.001 | 2.653 | 1.862 | 3.781 |
|  | Sex | -.601 | .173 | 12.045 | 1 | <.001 | .548 | .391 | .770 |
|  | obesity | .792 | .186 | 18.224 | 1 | <.001 | 2.208 | 1.535 | 3.177 |
|  | DM | .304 | .181 | 2.834 | 1 | .092 | 1.355 | .951 | 1.931 |
|  | IR | .741 | .162 | 20.968 | 1 | <.001 | 2.098 | 1.528 | 2.881 |
|  | HT | .358 | .187 | 3.676 | 1 | .055 | 1.430 | .992 | 2.061 |
|  | MetS | .891 | .210 | 17.944 | 1 | <.001 | 2.436 | 1.614 | 3.679 |
|  | High WC | 1.035 | .250 | 17.163 | 1 | <.001 | 2.816 | 1.725 | 4.595 |
|  | Constant | -3.589 | .394 | 83.089 | 1 | <.001 | .028 |  |  |
| a. Variable(s) entered on step 1: eduyr, marrital_status, income, alcohol, smoker, obesity, DM, IR, HT, Dyslipidemia, MetS, highWC. | | | | | | | | | |

**Assumption checks for models given in S4 Table:**

1. **Collinearity:**

| **Coefficients^a^** | | | |
| --- | --- | --- | --- |
| Model | | Collinearity Statistics | |
|  |  | Tolerance | VIF |
| 1 | site | .636 | 1.571 |
|  | age_cat | .762 | 1.311 |
|  | Education (years) | .716 | 1.397 |
|  | Marital status | .915 | 1.093 |
|  | income | .865 | 1.156 |
|  | alcohol | .659 | 1.518 |
|  | Sex | .766 | 1.305 |
|  | smoker | .910 | 1.099 |
|  | obesity | .603 | 1.659 |
|  | DM | .754 | 1.326 |
|  | IR | .808 | 1.238 |
|  | HT | .699 | 1.431 |
|  | Dyslipidemia | .858 | 1.166 |
|  | MetS | .423 | 2.366 |
|  | High WC | .487 | 2.053 |
| a. Dependent Variable: MASLD | | | |

1. **Influential outliers:**

| **Descriptive Statistics** | | | | | |
| --- | --- | --- | --- | --- | --- |
|  | N | Minimum | Maximum | Mean | Std. Deviation |
| Analog of Cook's influence statistics | 1039 | .00011 | .10542 | .0118163 | .01663002 |
| Standard residual | 1039 | -2.50222 | 2.55626 | .0381621 | .99119201 |
| Mahalanobis Distance | 1039 | 4.85649 | 29.43639 | 14.9855630 | 4.49582350 |
| Valid N (listwise) | 1039 |  |  |  |  |
