# Supplementary material for: Prevalence of MASLD and fibrosis in Turkey: Results from a multicenter study of at-risk populations
Source: PLoS One. 2026 Feb 12;21(2):e0341214. doi: 10.1371/journal.pone.0341214 (PMC12900293; doi:10.1371/journal.pone.0341214)
Supplement: S5 Table — (DOCX) [file pone.0341214.s005.docx]

**S5 Table. Multivariable logistic regression analyses of factors associated with the presence of significant fibrosis based on transient elastography**

|  | | B | S.E. | Wald | df | Sig. | Exp(B) | 95% C.I.for EXP(B) | |
| --- | --- | --- | --- | --- | --- | --- | --- | --- | --- |
|  |  |  |  |  |  |  |  | Lower | Upper |
| Step 1^a^ | Age category |  |  | 2.157 | 3 | .540 |  |  |  |
|  | age_cat(1) | -.295 | .462 | .408 | 1 | .523 | .744 | .301 | 1.841 |
|  | age_cat(2) | .026 | .469 | .003 | 1 | .956 | 1.026 | .409 | 2.571 |
|  | age_cat(3) | .223 | .523 | .182 | 1 | .670 | 1.250 | .448 | 3.485 |
|  | site | .058 | .291 | .039 | 1 | .843 | 1.060 | .598 | 1.876 |
|  | Sex | -.242 | .263 | .846 | 1 | .358 | .785 | .469 | 1.314 |
|  | Education (years) | -.010 | .028 | .131 | 1 | .718 | .990 | .938 | 1.045 |
|  | Marital status | .333 | .250 | 1.780 | 1 | .182 | 1.395 | .855 | 2.276 |
|  | Income level |  |  | 9.288 | 4 | .054 |  |  |  |
|  | income(1) | -1.132 | .714 | 2.510 | 1 | .113 | .322 | .079 | 1.308 |
|  | income(2) | -.591 | .653 | .821 | 1 | .365 | .553 | .154 | 1.990 |
|  | income(3) | -.646 | .670 | .930 | 1 | .335 | .524 | .141 | 1.948 |
|  | income(4) | -1.376 | .686 | 4.030 | 1 | .045 | .253 | .066 | .968 |
|  | alcohol | .010 | .308 | .001 | 1 | .974 | 1.010 | .552 | 1.848 |
|  | smoker | -.058 | .139 | .176 | 1 | .675 | .943 | .719 | 1.238 |
|  | obesity | 1.809 | .398 | 20.638 | 1 | <.001 | 6.102 | 2.796 | 13.317 |
|  | DM | .725 | .263 | 7.600 | 1 | .006 | 2.065 | 1.233 | 3.459 |
|  | IR | 1.429 | .346 | 17.071 | 1 | <.001 | 4.176 | 2.120 | 8.227 |
|  | HT | -.674 | .283 | 5.683 | 1 | .017 | .510 | .293 | .887 |
|  | Dyslipidemia | .265 | .382 | .482 | 1 | .487 | 1.304 | .617 | 2.755 |
|  | MetS | .867 | .397 | 4.770 | 1 | .029 | 2.380 | 1.093 | 5.183 |
|  | High WC | -.728 | .609 | 1.428 | 1 | .232 | .483 | .146 | 1.594 |
|  | Constant | -3.894 | 1.194 | 10.634 | 1 | .001 | .020 |  |  |
| Step 2^a^ | Age category |  |  | 2.159 | 3 | .540 |  |  |  |
|  | age_cat(1) | -.296 | .461 | .413 | 1 | .520 | .744 | .301 | 1.835 |
|  | age_cat(2) | .024 | .466 | .003 | 1 | .959 | 1.024 | .411 | 2.554 |
|  | age_cat(3) | .221 | .519 | .181 | 1 | .670 | 1.248 | .451 | 3.453 |
|  | site | .056 | .283 | .038 | 1 | .845 | 1.057 | .607 | 1.841 |
|  | Sex | -.244 | .253 | .930 | 1 | .335 | .784 | .477 | 1.286 |
|  | Education (years) | -.010 | .028 | .130 | 1 | .719 | .990 | .938 | 1.045 |
|  | Marital status | .334 | .249 | 1.793 | 1 | .181 | 1.396 | .857 | 2.275 |
|  | income |  |  | 9.348 | 4 | .053 |  |  |  |
|  | income(1) | -1.131 | .714 | 2.511 | 1 | .113 | .323 | .080 | 1.307 |
|  | income(2) | -.591 | .652 | .820 | 1 | .365 | .554 | .154 | 1.990 |
|  | income(3) | -.645 | .669 | .930 | 1 | .335 | .525 | .142 | 1.946 |
|  | income(4) | -1.374 | .683 | 4.047 | 1 | .044 | .253 | .066 | .965 |
|  | smoker | -.057 | .136 | .178 | 1 | .673 | .944 | .724 | 1.232 |
|  | obesity | 1.808 | .398 | 20.657 | 1 | <.001 | 6.099 | 2.797 | 13.302 |
|  | DM | .725 | .263 | 7.603 | 1 | .006 | 2.066 | 1.233 | 3.459 |
|  | IR | 1.430 | .346 | 17.105 | 1 | <.001 | 4.178 | 2.122 | 8.227 |
|  | HT | -.674 | .283 | 5.683 | 1 | .017 | .510 | .293 | .887 |
|  | Dyslipidemia | .265 | .382 | .483 | 1 | .487 | 1.304 | .617 | 2.755 |
|  | MetS | .867 | .397 | 4.774 | 1 | .029 | 2.379 | 1.093 | 5.175 |
|  | High WC | -.728 | .609 | 1.427 | 1 | .232 | .483 | .146 | 1.594 |
|  | Constant | -3.890 | 1.185 | 10.768 | 1 | .001 | .020 |  |  |
| Step 3^a^ | Age category |  |  | 2.201 | 3 | .532 |  |  |  |
|  | age_cat(1) | -.276 | .458 | .363 | 1 | .547 | .759 | .309 | 1.861 |
|  | age_cat(2) | .052 | .460 | .013 | 1 | .910 | 1.054 | .428 | 2.595 |
|  | age_cat(3) | .249 | .514 | .234 | 1 | .629 | 1.282 | .468 | 3.511 |
|  | site | .093 | .263 | .124 | 1 | .725 | 1.097 | .655 | 1.838 |
|  | Sex | -.236 | .252 | .881 | 1 | .348 | .789 | .482 | 1.293 |
|  | Marital status | .344 | .247 | 1.942 | 1 | .163 | 1.411 | .869 | 2.290 |
|  | Income level |  |  | 9.730 | 4 | .045 |  |  |  |
|  | income(1) | -1.121 | .711 | 2.484 | 1 | .115 | .326 | .081 | 1.314 |
|  | income(2) | -.590 | .650 | .824 | 1 | .364 | .554 | .155 | 1.982 |
|  | income(3) | -.663 | .665 | .995 | 1 | .319 | .515 | .140 | 1.896 |
|  | income(4) | -1.400 | .678 | 4.268 | 1 | .039 | .247 | .065 | .931 |
|  | smoker | -.056 | .135 | .170 | 1 | .680 | .946 | .725 | 1.233 |
|  | obesity | 1.813 | .398 | 20.777 | 1 | <.001 | 6.128 | 2.811 | 13.362 |
|  | DM | .734 | .262 | 7.848 | 1 | .005 | 2.083 | 1.247 | 3.481 |
|  | IR | 1.428 | .346 | 17.073 | 1 | <.001 | 4.172 | 2.119 | 8.215 |
|  | HT | -.674 | .283 | 5.688 | 1 | .017 | .510 | .293 | .887 |
|  | Dyslipidemia | .267 | .382 | .487 | 1 | .485 | 1.306 | .617 | 2.762 |
|  | MetS | .859 | .396 | 4.709 | 1 | .030 | 2.361 | 1.087 | 5.130 |
|  | High WC | -.715 | .608 | 1.382 | 1 | .240 | .489 | .149 | 1.611 |
|  | Constant | -4.104 | 1.025 | 16.036 | 1 | <.001 | .016 |  |  |
| Step 4^a^ | Age category |  |  | 2.338 | 3 | .505 |  |  |  |
|  | age_cat(1) | -.260 | .456 | .325 | 1 | .569 | .771 | .315 | 1.885 |
|  | age_cat(2) | .076 | .456 | .028 | 1 | .868 | 1.079 | .441 | 2.638 |
|  | age_cat(3) | .281 | .508 | .306 | 1 | .580 | 1.324 | .489 | 3.583 |
|  | site | .107 | .261 | .168 | 1 | .682 | 1.113 | .667 | 1.856 |
|  | Sex | -.233 | .252 | .857 | 1 | .354 | .792 | .484 | 1.297 |
|  | Marital status | .341 | .247 | 1.918 | 1 | .166 | 1.407 | .868 | 2.281 |
|  | Income level |  |  | 10.039 | 4 | .040 |  |  |  |
|  | income(1) | -1.129 | .710 | 2.533 | 1 | .112 | .323 | .080 | 1.299 |
|  | income(2) | -.600 | .649 | .857 | 1 | .355 | .549 | .154 | 1.956 |
|  | income(3) | -.682 | .662 | 1.062 | 1 | .303 | .505 | .138 | 1.850 |
|  | income(4) | -1.424 | .674 | 4.465 | 1 | .035 | .241 | .064 | .902 |
|  | obesity | 1.813 | .398 | 20.744 | 1 | <.001 | 6.130 | 2.809 | 13.376 |
|  | DM | .726 | .261 | 7.727 | 1 | .005 | 2.067 | 1.239 | 3.449 |
|  | IR | 1.429 | .346 | 17.105 | 1 | <.001 | 4.175 | 2.121 | 8.218 |
|  | HT | -.674 | .283 | 5.694 | 1 | .017 | .510 | .293 | .887 |
|  | Dyslipidemia | .269 | .382 | .493 | 1 | .483 | 1.308 | .618 | 2.768 |
|  | MetS | .873 | .395 | 4.891 | 1 | .027 | 2.395 | 1.104 | 5.193 |
|  | High WC | -.720 | .609 | 1.397 | 1 | .237 | .487 | .148 | 1.606 |
|  | Constant | -4.224 | .985 | 18.401 | 1 | <.001 | .015 |  |  |
| Step 5^a^ | Age category |  |  | 2.478 | 3 | .479 |  |  |  |
|  | age_cat(1) | -.246 | .455 | .292 | 1 | .589 | .782 | .321 | 1.908 |
|  | age_cat(2) | .108 | .453 | .056 | 1 | .812 | 1.114 | .458 | 2.707 |
|  | age_cat(3) | .308 | .506 | .371 | 1 | .542 | 1.361 | .505 | 3.668 |
|  | site | .102 | .260 | .153 | 1 | .695 | 1.107 | .665 | 1.844 |
|  | Sex | -.245 | .251 | .951 | 1 | .329 | .783 | .479 | 1.280 |
|  | Marital status | .350 | .246 | 2.019 | 1 | .155 | 1.419 | .876 | 2.298 |
|  | Income level |  |  | 10.154 | 4 | .038 |  |  |  |
|  | income(1) | -1.153 | .709 | 2.646 | 1 | .104 | .316 | .079 | 1.267 |
|  | income(2) | -.627 | .648 | .936 | 1 | .333 | .534 | .150 | 1.902 |
|  | income(3) | -.706 | .662 | 1.139 | 1 | .286 | .494 | .135 | 1.805 |
|  | income(4) | -1.450 | .673 | 4.636 | 1 | .031 | .235 | .063 | .878 |
|  | obesity | 1.818 | .398 | 20.849 | 1 | <.001 | 6.161 | 2.823 | 13.445 |
|  | DM | .723 | .261 | 7.673 | 1 | .006 | 2.060 | 1.235 | 3.435 |
|  | IR | 1.444 | .345 | 17.536 | 1 | <.001 | 4.239 | 2.156 | 8.334 |
|  | HT | -.702 | .280 | 6.280 | 1 | .012 | .496 | .286 | .858 |
|  | MetS | .947 | .381 | 6.167 | 1 | .013 | 2.578 | 1.221 | 5.445 |
|  | High WC | -.769 | .604 | 1.621 | 1 | .203 | .463 | .142 | 1.514 |
|  | Constant | -3.992 | .924 | 18.680 | 1 | <.001 | .018 |  |  |
| Step 6^a^ | Age category |  |  | 2.370 | 3 | .499 |  |  |  |
|  | age_cat(1) | -.242 | .454 | .284 | 1 | .594 | .785 | .323 | 1.911 |
|  | age_cat(2) | .093 | .451 | .043 | 1 | .837 | 1.097 | .453 | 2.656 |
|  | age_cat(3) | .303 | .504 | .361 | 1 | .548 | 1.353 | .504 | 3.634 |
|  | site | .109 | .260 | .177 | 1 | .674 | 1.116 | .671 | 1.856 |
|  | Sex | -.248 | .250 | .984 | 1 | .321 | .780 | .478 | 1.274 |
|  | Marital status | .345 | .246 | 1.971 | 1 | .160 | 1.412 | .872 | 2.286 |
|  | Income level |  |  | 9.892 | 4 | .042 |  |  |  |
|  | income(1) | -1.114 | .704 | 2.508 | 1 | .113 | .328 | .083 | 1.303 |
|  | income(2) | -.608 | .643 | .896 | 1 | .344 | .544 | .155 | 1.918 |
|  | income(3) | -.661 | .655 | 1.016 | 1 | .314 | .517 | .143 | 1.866 |
|  | income(4) | -1.414 | .668 | 4.478 | 1 | .034 | .243 | .066 | .901 |
|  | obesity | 1.588 | .330 | 23.216 | 1 | <.001 | 4.896 | 2.566 | 9.342 |
|  | DM | .748 | .260 | 8.259 | 1 | .004 | 2.114 | 1.269 | 3.521 |
|  | IR | 1.436 | .344 | 17.392 | 1 | <.001 | 4.202 | 2.140 | 8.249 |
|  | HT | -.665 | .280 | 5.654 | 1 | .017 | .514 | .297 | .890 |
|  | MetS | .772 | .349 | 4.905 | 1 | .027 | 2.164 | 1.093 | 4.286 |
|  | Constant | -4.438 | .868 | 26.169 | 1 | <.001 | .012 |  |  |
| Step 7^a^ | Age category |  |  | 2.687 | 3 | .442 |  |  |  |
|  | age_cat(1) | -.124 | .446 | .077 | 1 | .782 | .884 | .369 | 2.118 |
|  | age_cat(2) | .233 | .440 | .281 | 1 | .596 | 1.263 | .533 | 2.994 |
|  | age_cat(3) | .447 | .494 | .819 | 1 | .366 | 1.563 | .594 | 4.116 |
|  | site | .102 | .258 | .155 | 1 | .694 | 1.107 | .667 | 1.836 |
|  | Sex | -.239 | .249 | .920 | 1 | .338 | .788 | .483 | 1.283 |
|  | Income level |  |  | 9.560 | 4 | .049 |  |  |  |
|  | income(1) | -1.024 | .700 | 2.143 | 1 | .143 | .359 | .091 | 1.415 |
|  | income(2) | -.549 | .641 | .734 | 1 | .392 | .577 | .164 | 2.028 |
|  | income(3) | -.616 | .655 | .885 | 1 | .347 | .540 | .150 | 1.949 |
|  | income(4) | -1.361 | .667 | 4.165 | 1 | .041 | .256 | .069 | .948 |
|  | obesity | 1.578 | .330 | 22.930 | 1 | <.001 | 4.845 | 2.540 | 9.242 |
|  | DM | .775 | .259 | 8.944 | 1 | .003 | 2.172 | 1.306 | 3.610 |
|  | IR | 1.436 | .344 | 17.464 | 1 | <.001 | 4.205 | 2.144 | 8.246 |
|  | HT | -.625 | .278 | 5.058 | 1 | .025 | .535 | .310 | .923 |
|  | MetS | .720 | .346 | 4.326 | 1 | .038 | 2.053 | 1.042 | 4.045 |
|  | Constant | -4.263 | .861 | 24.498 | 1 | <.001 | .014 |  |  |
| a. Variable(s) entered on step 1: eduyr, marrital_status, income, alcohol, smoker, obesity, DM, IR, HT, Dyslipidemia, MetS, highWC. | | | | | | | | | |

**Assumption checks for the models given in S5 Table:**

1. **Collinearity:**

| **Coefficients^a^** | | | |
| --- | --- | --- | --- |
| Model | | Collinearity Statistics | |
|  |  | Tolerance | VIF |
| 1 | site | .636 | 1.571 |
|  | Age category | .762 | 1.311 |
|  | Education (years) | .716 | 1.397 |
|  | Marital status | .915 | 1.093 |
|  | income | .865 | 1.156 |
|  | alcohol | .659 | 1.518 |
|  | Sex | .766 | 1.305 |
|  | smoker | .910 | 1.099 |
|  | obesity | .603 | 1.659 |
|  | DM | .754 | 1.326 |
|  | IR | .808 | 1.238 |
|  | HT | .699 | 1.431 |
|  | Dyslipidemia | .858 | 1.166 |
|  | MetS | .423 | 2.366 |
|  | High WC | .487 | 2.053 |
| a. Dependent Variable: FIBROSIS | | | |

1. **Influential outliers:**

| **Descriptive Statistics** | | | | | |
| --- | --- | --- | --- | --- | --- |
|  | N | Minimum | Maximum | Mean | Std. Deviation |
| Analog of Cook's influence statistics | 1039 | .00000 | .47503 | .0147580 | .04350990 |
| Standard residual | 1039 | -1.38387 | 3.15369 | -.1411381 | .71591148 |
| Mahalanobis Distance | 1039 | 4.85649 | 29.43639 | 14.9855630 | 4.49582350 |
| Valid N (listwise) | 1039 |  |  |  |  |
