# Supplementary material for: Prevalence of MASLD and fibrosis in Turkey: Results from a multicenter study of at-risk populations
Source: PLoS One. 2026 Feb 12;21(2):e0341214. doi: 10.1371/journal.pone.0341214 (PMC12900293; doi:10.1371/journal.pone.0341214)
Supplement: S6 Table — (DOCX) [file pone.0341214.s006.docx]

**S6 Table. Sensitivity analyses without insulin resistance for factors associated with the presence of MASLD based on transient elastography**

| **Variables in the Equation** | | | | | | | | | |
| --- | --- | --- | --- | --- | --- | --- | --- | --- | --- |
|  | | B | S.E. | Wald | df | Sig. | Exp(B) | 95% C.I.for EXP(B) | |
|  |  |  |  |  |  |  |  | Lower | Upper |
| Step 1^a^ | site | 1.099 | .198 | 30.904 | 1 | <.001 | 3.000 | 2.037 | 4.419 |
|  | Age category |  |  | 3.253 | 3 | .354 |  |  |  |
|  | age_cat(1) | .476 | .284 | 2.812 | 1 | .094 | 1.610 | .923 | 2.808 |
|  | age_cat(2) | .496 | .289 | 2.945 | 1 | .086 | 1.642 | .932 | 2.892 |
|  | age_cat(3) | .450 | .344 | 1.715 | 1 | .190 | 1.569 | .800 | 3.079 |
|  | Sex | -.630 | .183 | 11.884 | 1 | <.001 | .533 | .372 | .762 |
|  | Education level |  |  | .953 | 4 | .917 |  |  |  |
|  | education(1) | -.060 | .327 | .033 | 1 | .855 | .942 | .496 | 1.789 |
|  | education(2) | -.143 | .255 | .314 | 1 | .575 | .867 | .525 | 1.429 |
|  | education(3) | .028 | .260 | .011 | 1 | .915 | 1.028 | .618 | 1.710 |
|  | education(4) | -.145 | .358 | .165 | 1 | .685 | .865 | .429 | 1.744 |
|  | Marital status |  |  | .047 | 2 | .977 |  |  |  |
|  | marrital_status(1) | -.024 | .226 | .011 | 1 | .915 | .976 | .627 | 1.520 |
|  | marrital_status(2) | .027 | .316 | .007 | 1 | .933 | 1.027 | .553 | 1.908 |
|  | Income level |  |  | 6.167 | 4 | .187 |  |  |  |
|  | income(1) | 1.049 | .579 | 3.280 | 1 | .070 | 2.855 | .917 | 8.887 |
|  | income(2) | .663 | .550 | 1.452 | 1 | .228 | 1.940 | .660 | 5.699 |
|  | income(3) | .470 | .560 | .704 | 1 | .401 | 1.600 | .534 | 4.800 |
|  | income(4) | .755 | .557 | 1.838 | 1 | .175 | 2.127 | .714 | 6.335 |
|  | alcohol | .072 | .196 | .136 | 1 | .712 | 1.075 | .732 | 1.579 |
|  | current smoker | .074 | .179 | .169 | 1 | .681 | 1.076 | .757 | 1.530 |
|  | obesity | .902 | .186 | 23.640 | 1 | <.001 | 2.465 | 1.714 | 3.547 |
|  | DM | .356 | .180 | 3.909 | 1 | .048 | 1.428 | 1.003 | 2.033 |
|  | HT | .427 | .188 | 5.150 | 1 | .023 | 1.532 | 1.060 | 2.215 |
|  | Dyslipidemia | .306 | .215 | 2.023 | 1 | .155 | 1.357 | .891 | 2.068 |
|  | MetS | .950 | .219 | 18.733 | 1 | <.001 | 2.585 | 1.681 | 3.973 |
|  | High WC | 1.120 | .256 | 19.186 | 1 | <.001 | 3.065 | 1.857 | 5.060 |
|  | Constant | -4.403 | .796 | 30.603 | 1 | <.001 | .012 |  |  |
| Step 2^a^ | site | 1.095 | .197 | 31.029 | 1 | <.001 | 2.988 | 2.033 | 4.393 |
|  | Age category |  |  | 3.321 | 3 | .345 |  |  |  |
|  | age_cat(1) | .472 | .279 | 2.856 | 1 | .091 | 1.603 | .927 | 2.769 |
|  | age_cat(2) | .492 | .284 | 2.996 | 1 | .083 | 1.635 | .937 | 2.854 |
|  | age_cat(3) | .444 | .338 | 1.728 | 1 | .189 | 1.560 | .804 | 3.026 |
|  | Sex | -.628 | .182 | 11.855 | 1 | <.001 | .534 | .373 | .763 |
|  | Education level |  |  | .961 | 4 | .916 |  |  |  |
|  | education(1) | -.061 | .327 | .034 | 1 | .853 | .941 | .496 | 1.787 |
|  | education(2) | -.143 | .254 | .315 | 1 | .575 | .867 | .527 | 1.427 |
|  | education(3) | .029 | .255 | .013 | 1 | .910 | 1.029 | .624 | 1.698 |
|  | education(4) | -.143 | .356 | .162 | 1 | .687 | .866 | .431 | 1.740 |
|  | Income level |  |  | 6.177 | 4 | .186 |  |  |  |
|  | income(1) | 1.048 | .579 | 3.279 | 1 | .070 | 2.852 | .917 | 8.867 |
|  | income(2) | .663 | .549 | 1.455 | 1 | .228 | 1.940 | .661 | 5.695 |
|  | income(3) | .469 | .560 | .701 | 1 | .402 | 1.598 | .533 | 4.785 |
|  | income(4) | .754 | .556 | 1.841 | 1 | .175 | 2.126 | .715 | 6.322 |
|  | alcohol | .070 | .196 | .129 | 1 | .720 | 1.073 | .731 | 1.574 |
|  | current smoker | .075 | .179 | .177 | 1 | .674 | 1.078 | .759 | 1.532 |
|  | obesity | .901 | .185 | 23.629 | 1 | <.001 | 2.463 | 1.712 | 3.542 |
|  | DM | .357 | .180 | 3.938 | 1 | .047 | 1.429 | 1.004 | 2.034 |
|  | HT | .427 | .188 | 5.158 | 1 | .023 | 1.532 | 1.060 | 2.214 |
|  | Dyslipidemia | .308 | .214 | 2.064 | 1 | .151 | 1.361 | .894 | 2.071 |
|  | MetS | .950 | .219 | 18.806 | 1 | <.001 | 2.585 | 1.683 | 3.970 |
|  | High WC | 1.118 | .255 | 19.184 | 1 | <.001 | 3.059 | 1.855 | 5.046 |
|  | Constant | -4.409 | .781 | 31.840 | 1 | <.001 | .012 |  |  |
| Step 3^a^ | site | 1.097 | .188 | 34.071 | 1 | <.001 | 2.995 | 2.072 | 4.328 |
|  | Age category |  |  | 3.347 | 3 | .341 |  |  |  |
|  | age_cat(1) | .464 | .277 | 2.807 | 1 | .094 | 1.590 | .924 | 2.736 |
|  | age_cat(2) | .492 | .281 | 3.072 | 1 | .080 | 1.636 | .943 | 2.836 |
|  | age_cat(3) | .443 | .335 | 1.752 | 1 | .186 | 1.558 | .808 | 3.004 |
|  | Sex | -.624 | .182 | 11.759 | 1 | <.001 | .536 | .375 | .766 |
|  | Income level |  |  | 6.774 | 4 | .148 |  |  |  |
|  | income(1) | 1.109 | .573 | 3.742 | 1 | .053 | 3.031 | .985 | 9.322 |
|  | income(2) | .710 | .545 | 1.696 | 1 | .193 | 2.035 | .698 | 5.926 |
|  | income(3) | .519 | .555 | .872 | 1 | .350 | 1.680 | .566 | 4.987 |
|  | income(4) | .812 | .550 | 2.175 | 1 | .140 | 2.251 | .766 | 6.620 |
|  | alcohol | .080 | .195 | .168 | 1 | .682 | 1.083 | .739 | 1.586 |
|  | current smoker | .073 | .179 | .168 | 1 | .682 | 1.076 | .758 | 1.528 |
|  | obesity | .909 | .185 | 24.112 | 1 | <.001 | 2.482 | 1.727 | 3.567 |
|  | DM | .356 | .179 | 3.940 | 1 | .047 | 1.428 | 1.004 | 2.030 |
|  | HT | .429 | .187 | 5.264 | 1 | .022 | 1.536 | 1.065 | 2.216 |
|  | Dyslipidemia | .297 | .213 | 1.937 | 1 | .164 | 1.346 | .886 | 2.044 |
|  | MetS | .947 | .217 | 18.973 | 1 | <.001 | 2.578 | 1.684 | 3.948 |
|  | High WC | 1.125 | .253 | 19.829 | 1 | <.001 | 3.080 | 1.877 | 5.054 |
|  | Constant | -4.514 | .708 | 40.595 | 1 | <.001 | .011 |  |  |
| Step 4^a^ | site | 1.076 | .181 | 35.477 | 1 | <.001 | 2.932 | 2.058 | 4.178 |
|  | Age category |  |  | 3.252 | 3 | .354 |  |  |  |
|  | age_cat(1) | .456 | .276 | 2.724 | 1 | .099 | 1.578 | .918 | 2.712 |
|  | age_cat(2) | .483 | .280 | 2.980 | 1 | .084 | 1.622 | .937 | 2.807 |
|  | age_cat(3) | .428 | .333 | 1.655 | 1 | .198 | 1.534 | .799 | 2.945 |
|  | Sex | -.643 | .176 | 13.394 | 1 | <.001 | .526 | .373 | .742 |
|  | Income level |  |  | 6.739 | 4 | .150 |  |  |  |
|  | income(1) | 1.112 | .573 | 3.770 | 1 | .052 | 3.040 | .990 | 9.340 |
|  | income(2) | .720 | .544 | 1.751 | 1 | .186 | 2.055 | .707 | 5.974 |
|  | income(3) | .535 | .553 | .933 | 1 | .334 | 1.707 | .577 | 5.049 |
|  | income(4) | .832 | .548 | 2.312 | 1 | .128 | 2.299 | .786 | 6.723 |
|  | current smoker | .091 | .174 | .271 | 1 | .603 | 1.095 | .778 | 1.540 |
|  | obesity | .901 | .184 | 23.984 | 1 | <.001 | 2.461 | 1.716 | 3.529 |
|  | DM | .354 | .179 | 3.904 | 1 | .048 | 1.425 | 1.003 | 2.026 |
|  | HT | .429 | .187 | 5.275 | 1 | .022 | 1.536 | 1.065 | 2.217 |
|  | Dyslipidemia | .294 | .213 | 1.901 | 1 | .168 | 1.342 | .883 | 2.038 |
|  | MetS | .943 | .217 | 18.867 | 1 | <.001 | 2.567 | 1.678 | 3.929 |
|  | High WC | 1.129 | .253 | 19.980 | 1 | <.001 | 3.091 | 1.885 | 5.071 |
|  | Constant | -4.447 | .689 | 41.690 | 1 | <.001 | .012 |  |  |
| Step 5^a^ | site | 1.072 | .180 | 35.292 | 1 | <.001 | 2.921 | 2.051 | 4.160 |
|  | Age category |  |  | 3.107 | 3 | .375 |  |  |  |
|  | age_cat(1) | .447 | .276 | 2.631 | 1 | .105 | 1.564 | .911 | 2.683 |
|  | age_cat(2) | .466 | .278 | 2.814 | 1 | .093 | 1.594 | .925 | 2.747 |
|  | age_cat(3) | .402 | .329 | 1.494 | 1 | .222 | 1.494 | .785 | 2.846 |
|  | Sex | -.647 | .176 | 13.608 | 1 | <.001 | .523 | .371 | .738 |
|  | Income level |  |  | 6.691 | 4 | .153 |  |  |  |
|  | income(1) | 1.110 | .574 | 3.740 | 1 | .053 | 3.035 | .985 | 9.349 |
|  | income(2) | .720 | .546 | 1.741 | 1 | .187 | 2.055 | .705 | 5.991 |
|  | income(3) | .538 | .555 | .940 | 1 | .332 | 1.712 | .577 | 5.080 |
|  | income(4) | .836 | .549 | 2.322 | 1 | .128 | 2.308 | .787 | 6.769 |
|  | obesity | .900 | .184 | 23.989 | 1 | <.001 | 2.461 | 1.716 | 3.528 |
|  | DM | .357 | .179 | 3.976 | 1 | .046 | 1.430 | 1.006 | 2.032 |
|  | HT | .428 | .187 | 5.238 | 1 | .022 | 1.534 | 1.063 | 2.213 |
|  | Dyslipidemia | .292 | .213 | 1.877 | 1 | .171 | 1.339 | .882 | 2.033 |
|  | MetS | .937 | .217 | 18.693 | 1 | <.001 | 2.553 | 1.669 | 3.904 |
|  | High WC | 1.129 | .253 | 19.983 | 1 | <.001 | 3.092 | 1.885 | 5.072 |
|  | Constant | -4.395 | .682 | 41.508 | 1 | <.001 | .012 |  |  |
| Step 6^a^ | site | 1.078 | .180 | 35.777 | 1 | <.001 | 2.938 | 2.064 | 4.182 |
|  | Age category |  |  | 3.985 | 3 | .263 |  |  |  |
|  | age_cat(1) | .491 | .272 | 3.246 | 1 | .072 | 1.634 | .958 | 2.786 |
|  | age_cat(2) | .525 | .273 | 3.695 | 1 | .055 | 1.691 | .990 | 2.888 |
|  | age_cat(3) | .476 | .323 | 2.169 | 1 | .141 | 1.610 | .854 | 3.035 |
|  | Sex | -.672 | .174 | 14.870 | 1 | <.001 | .511 | .363 | .719 |
|  | Income level |  |  | 6.378 | 4 | .173 |  |  |  |
|  | income(1) | 1.071 | .573 | 3.496 | 1 | .062 | 2.918 | .950 | 8.964 |
|  | income(2) | .687 | .545 | 1.589 | 1 | .208 | 1.987 | .683 | 5.779 |
|  | income(3) | .515 | .554 | .864 | 1 | .353 | 1.673 | .565 | 4.957 |
|  | income(4) | .812 | .548 | 2.194 | 1 | .139 | 2.252 | .769 | 6.593 |
|  | obesity | .907 | .184 | 24.361 | 1 | <.001 | 2.477 | 1.728 | 3.552 |
|  | DM | .345 | .179 | 3.725 | 1 | .054 | 1.412 | .995 | 2.005 |
|  | HT | .400 | .185 | 4.661 | 1 | .031 | 1.492 | 1.038 | 2.146 |
|  | MetS | 1.025 | .207 | 24.496 | 1 | <.001 | 2.787 | 1.857 | 4.183 |
|  | High WC | 1.074 | .248 | 18.731 | 1 | <.001 | 2.926 | 1.799 | 4.757 |
|  | Constant | -4.161 | .657 | 40.163 | 1 | <.001 | .016 |  |  |
| Step 7^a^ | site | 1.056 | .178 | 35.302 | 1 | <.001 | 2.875 | 2.029 | 4.073 |
|  | Age category |  |  | 4.093 | 3 | .252 |  |  |  |
|  | age_cat(1) | .493 | .270 | 3.323 | 1 | .068 | 1.637 | .964 | 2.782 |
|  | age_cat(2) | .529 | .271 | 3.813 | 1 | .051 | 1.697 | .998 | 2.885 |
|  | age_cat(3) | .472 | .321 | 2.160 | 1 | .142 | 1.604 | .854 | 3.012 |
|  | Sex | -.674 | .170 | 15.629 | 1 | <.001 | .510 | .365 | .712 |
|  | obesity | .884 | .183 | 23.421 | 1 | <.001 | 2.421 | 1.693 | 3.464 |
|  | DM | .355 | .178 | 3.975 | 1 | .046 | 1.426 | 1.006 | 2.021 |
|  | HT | .387 | .184 | 4.411 | 1 | .036 | 1.472 | 1.026 | 2.111 |
|  | MetS | 1.013 | .206 | 24.237 | 1 | <.001 | 2.753 | 1.839 | 4.120 |
|  | highWC | 1.074 | .246 | 19.023 | 1 | <.001 | 2.928 | 1.807 | 4.745 |
|  | Constant | -3.394 | .385 | 77.890 | 1 | <.001 | .034 |  |  |
| a. Variable(s) entered on step 1: education, marrital_status, income, alcohol, current smoker, obesity, DM, HT, Dyslipidemia, MetS, highWC. | | | | | | | | | |
