# Supplementary material for: Prevalence of MASLD and fibrosis in Turkey: Results from a multicenter study of at-risk populations
Source: PLoS One. 2026 Feb 12;21(2):e0341214. doi: 10.1371/journal.pone.0341214 (PMC12900293; doi:10.1371/journal.pone.0341214)
Supplement: S7 Table — (DOCX) [file pone.0341214.s007.docx]

**S7 Table. Sensitivity analyses without insulin resistance for factors associated with the presence of significant fibrosis based on transient elastography**

| **Variables in the Equation** | | | | | | | | | |
| --- | --- | --- | --- | --- | --- | --- | --- | --- | --- |
|  | | B | S.E. | Wald | df | Sig. | Exp(B) | 95% C.I.for EXP(B) | |
|  |  |  |  |  |  |  |  | Lower | Upper |
| Step 1^a^ | site | .298 | .289 | 1.062 | 1 | .303 | 1.347 | .764 | 2.373 |
|  | Age category |  |  | 1.567 | 3 | .667 |  |  |  |
|  | age_cat(1) | -.146 | .466 | .098 | 1 | .754 | .864 | .347 | 2.153 |
|  | age_cat(2) | .102 | .472 | .047 | 1 | .829 | 1.107 | .439 | 2.796 |
|  | age_cat(3) | .311 | .527 | .349 | 1 | .555 | 1.365 | .486 | 3.835 |
|  | gender | -.338 | .263 | 1.642 | 1 | .200 | .714 | .426 | 1.196 |
|  | Education level |  |  | 4.595 | 4 | .331 |  |  |  |
|  | education(1) | -.823 | .469 | 3.077 | 1 | .079 | .439 | .175 | 1.101 |
|  | education(2) | -.274 | .323 | .720 | 1 | .396 | .760 | .403 | 1.432 |
|  | education(3) | -.348 | .352 | .976 | 1 | .323 | .706 | .355 | 1.408 |
|  | education(4) | .197 | .510 | .148 | 1 | .700 | 1.217 | .448 | 3.308 |
|  | Marital status |  |  | 3.811 | 2 | .149 |  |  |  |
|  | marrital_status(1) | .000 | .355 | .000 | 1 | 1.000 | 1.000 | .499 | 2.006 |
|  | marrital_status(2) | .661 | .458 | 2.081 | 1 | .149 | 1.937 | .789 | 4.756 |
|  | Income level |  |  | 9.534 | 4 | .049 |  |  |  |
|  | income(1) | -1.080 | .717 | 2.270 | 1 | .132 | .340 | .083 | 1.384 |
|  | income(2) | -.471 | .654 | .520 | 1 | .471 | .624 | .173 | 2.247 |
|  | income(3) | -.541 | .671 | .649 | 1 | .420 | .582 | .156 | 2.170 |
|  | income(4) | -1.284 | .689 | 3.475 | 1 | .062 | .277 | .072 | 1.068 |
|  | alcohol | .121 | .305 | .156 | 1 | .692 | 1.128 | .620 | 2.052 |
|  | current smoker | -.104 | .274 | .145 | 1 | .703 | .901 | .527 | 1.540 |
|  | obesity | 1.978 | .402 | 24.195 | 1 | <.001 | 7.227 | 3.286 | 15.894 |
|  | DM | .737 | .261 | 7.969 | 1 | .005 | 2.089 | 1.253 | 3.485 |
|  | HT | -.637 | .281 | 5.139 | 1 | .023 | .529 | .305 | .917 |
|  | Dyslipidemia | .441 | .379 | 1.353 | 1 | .245 | 1.555 | .739 | 3.271 |
|  | MetS | 1.082 | .393 | 7.567 | 1 | .006 | 2.950 | 1.365 | 6.377 |
|  | highWC | -.694 | .614 | 1.276 | 1 | .259 | .500 | .150 | 1.666 |
|  | Constant | -3.534 | 1.135 | 9.698 | 1 | .002 | .029 |  |  |
| Step 2^a^ | site | .300 | .289 | 1.081 | 1 | .299 | 1.350 | .766 | 2.380 |
|  | Age category |  |  | 1.674 | 3 | .643 |  |  |  |
|  | age_cat(1) | -.136 | .465 | .085 | 1 | .770 | .873 | .351 | 2.170 |
|  | age_cat(2) | .117 | .471 | .062 | 1 | .804 | 1.124 | .447 | 2.826 |
|  | age_cat(3) | .334 | .523 | .409 | 1 | .523 | 1.397 | .501 | 3.895 |
|  | Sex | -.340 | .263 | 1.665 | 1 | .197 | .712 | .425 | 1.193 |
|  | Education level |  |  | 4.672 | 4 | .323 |  |  |  |
|  | education(1) | -.830 | .469 | 3.131 | 1 | .077 | .436 | .174 | 1.093 |
|  | education(2) | -.277 | .323 | .733 | 1 | .392 | .758 | .402 | 1.429 |
|  | education(3) | -.339 | .351 | .934 | 1 | .334 | .712 | .358 | 1.417 |
|  | education(4) | .208 | .509 | .166 | 1 | .683 | 1.231 | .454 | 3.336 |
|  | Marital status |  |  | 3.793 | 2 | .150 |  |  |  |
|  | marrital_status(1) | -.003 | .355 | .000 | 1 | .994 | .997 | .498 | 1.999 |
|  | marrital_status(2) | .657 | .458 | 2.059 | 1 | .151 | 1.928 | .787 | 4.727 |
|  | Income level |  |  | 9.727 | 4 | .045 |  |  |  |
|  | income(1) | -1.080 | .716 | 2.271 | 1 | .132 | .340 | .083 | 1.383 |
|  | income(2) | -.472 | .653 | .523 | 1 | .470 | .624 | .173 | 2.244 |
|  | income(3) | -.551 | .670 | .676 | 1 | .411 | .576 | .155 | 2.144 |
|  | income(4) | -1.299 | .688 | 3.567 | 1 | .059 | .273 | .071 | 1.050 |
|  | alcohol | .095 | .298 | .102 | 1 | .749 | 1.100 | .614 | 1.971 |
|  | obesity | 1.978 | .402 | 24.161 | 1 | <.001 | 7.226 | 3.284 | 15.900 |
|  | DM | .732 | .261 | 7.888 | 1 | .005 | 2.080 | 1.248 | 3.468 |
|  | HT | -.637 | .281 | 5.132 | 1 | .023 | .529 | .305 | .918 |
|  | Dyslipidemia | .445 | .379 | 1.374 | 1 | .241 | 1.560 | .742 | 3.282 |
|  | MetS | 1.092 | .393 | 7.733 | 1 | .005 | 2.981 | 1.380 | 6.437 |
|  | High WC | -.701 | .615 | 1.298 | 1 | .255 | .496 | .149 | 1.656 |
|  | Constant | -3.567 | 1.133 | 9.907 | 1 | .002 | .028 |  |  |
| Step 3^a^ | site | .278 | .280 | .985 | 1 | .321 | 1.320 | .763 | 2.283 |
|  | Age category |  |  | 1.616 | 3 | .656 |  |  |  |
|  | age_cat(1) | -.152 | .462 | .108 | 1 | .742 | .859 | .348 | 2.123 |
|  | age_cat(2) | .097 | .466 | .043 | 1 | .835 | 1.102 | .442 | 2.745 |
|  | age_cat(3) | .308 | .516 | .356 | 1 | .551 | 1.361 | .495 | 3.742 |
|  | Sex | -.361 | .254 | 2.025 | 1 | .155 | .697 | .424 | 1.146 |
|  | Education level |  |  | 4.598 | 4 | .331 |  |  |  |
|  | education(1) | -.824 | .468 | 3.096 | 1 | .079 | .439 | .175 | 1.098 |
|  | education(2) | -.273 | .323 | .715 | 1 | .398 | .761 | .404 | 1.433 |
|  | education(3) | -.327 | .349 | .879 | 1 | .348 | .721 | .364 | 1.428 |
|  | education(4) | .209 | .509 | .168 | 1 | .682 | 1.232 | .454 | 3.342 |
|  | Marital status |  |  | 3.794 | 2 | .150 |  |  |  |
|  | marrital_status(1) | .005 | .354 | .000 | 1 | .989 | 1.005 | .502 | 2.011 |
|  | marrital_status(2) | .663 | .457 | 2.102 | 1 | .147 | 1.940 | .792 | 4.753 |
|  | income |  |  | 9.627 | 4 | .047 |  |  |  |
|  | income(1) | -1.072 | .716 | 2.243 | 1 | .134 | .342 | .084 | 1.392 |
|  | income(2) | -.466 | .653 | .509 | 1 | .476 | .628 | .175 | 2.257 |
|  | income(3) | -.540 | .669 | .651 | 1 | .420 | .583 | .157 | 2.164 |
|  | income(4) | -1.281 | .685 | 3.494 | 1 | .062 | .278 | .072 | 1.064 |
|  | obesity | 1.970 | .402 | 24.064 | 1 | <.001 | 7.169 | 3.263 | 15.748 |
|  | DM | .735 | .261 | 7.944 | 1 | .005 | 2.085 | 1.251 | 3.474 |
|  | HT | -.636 | .281 | 5.129 | 1 | .024 | .529 | .305 | .918 |
|  | Dyslipidemia | .444 | .379 | 1.373 | 1 | .241 | 1.560 | .742 | 3.280 |
|  | MetS | 1.086 | .392 | 7.666 | 1 | .006 | 2.963 | 1.373 | 6.391 |
|  | highWC | -.695 | .615 | 1.277 | 1 | .258 | .499 | .150 | 1.666 |
|  | Constant | -3.489 | 1.107 | 9.941 | 1 | .002 | .031 |  |  |
| Step 4^a^ | site | .314 | .256 | 1.501 | 1 | .221 | 1.369 | .828 | 2.262 |
|  | Age category |  |  | 1.731 | 3 | .630 |  |  |  |
|  | age_cat(1) | -.133 | .457 | .085 | 1 | .770 | .875 | .357 | 2.142 |
|  | age_cat(2) | .152 | .457 | .111 | 1 | .739 | 1.164 | .475 | 2.850 |
|  | age_cat(3) | .328 | .508 | .418 | 1 | .518 | 1.388 | .513 | 3.756 |
|  | Sex | -.334 | .249 | 1.798 | 1 | .180 | .716 | .440 | 1.167 |
|  | Marital status |  |  | 3.722 | 2 | .156 |  |  |  |
|  | marrital_status(1) | .023 | .346 | .005 | 1 | .946 | 1.024 | .519 | 2.018 |
|  | marrital_status(2) | .669 | .451 | 2.199 | 1 | .138 | 1.952 | .806 | 4.728 |
|  | Income level |  |  | 9.546 | 4 | .049 |  |  |  |
|  | income(1) | -1.075 | .700 | 2.356 | 1 | .125 | .341 | .087 | 1.346 |
|  | income(2) | -.499 | .639 | .610 | 1 | .435 | .607 | .173 | 2.125 |
|  | income(3) | -.553 | .655 | .713 | 1 | .398 | .575 | .159 | 2.076 |
|  | income(4) | -1.284 | .667 | 3.707 | 1 | .054 | .277 | .075 | 1.023 |
|  | obesity | 1.969 | .400 | 24.258 | 1 | <.001 | 7.165 | 3.272 | 15.686 |
|  | DM | .760 | .258 | 8.651 | 1 | .003 | 2.137 | 1.288 | 3.546 |
|  | HT | -.636 | .279 | 5.209 | 1 | .022 | .529 | .307 | .914 |
|  | Dyslipidemia | .389 | .376 | 1.073 | 1 | .300 | 1.476 | .706 | 3.084 |
|  | MetS | 1.078 | .390 | 7.629 | 1 | .006 | 2.939 | 1.368 | 6.314 |
|  | High WC | -.657 | .613 | 1.145 | 1 | .285 | .519 | .156 | 1.726 |
|  | Constant | -3.817 | .972 | 15.422 | 1 | <.001 | .022 |  |  |
| Step 5^a^ | site | .322 | .256 | 1.585 | 1 | .208 | 1.380 | .836 | 2.281 |
|  | Age category |  |  | 1.623 | 3 | .654 |  |  |  |
|  | age_cat(1) | -.138 | .455 | .092 | 1 | .762 | .871 | .357 | 2.126 |
|  | age_cat(2) | .129 | .454 | .081 | 1 | .776 | 1.138 | .467 | 2.771 |
|  | age_cat(3) | .311 | .505 | .379 | 1 | .538 | 1.365 | .507 | 3.674 |
|  | Sex | -.336 | .248 | 1.825 | 1 | .177 | .715 | .439 | 1.163 |
|  | Marital status |  |  | 3.721 | 2 | .156 |  |  |  |
|  | marrital_status(1) | .007 | .345 | .000 | 1 | .983 | 1.007 | .513 | 1.980 |
|  | marrital_status(2) | .656 | .450 | 2.120 | 1 | .145 | 1.926 | .797 | 4.656 |
|  | Income level |  |  | 9.248 | 4 | .055 |  |  |  |
|  | income(1) | -1.037 | .696 | 2.221 | 1 | .136 | .354 | .091 | 1.386 |
|  | income(2) | -.477 | .635 | .563 | 1 | .453 | .621 | .179 | 2.156 |
|  | income(3) | -.507 | .650 | .609 | 1 | .435 | .602 | .169 | 2.153 |
|  | income(4) | -1.241 | .662 | 3.514 | 1 | .061 | .289 | .079 | 1.058 |
|  | obesity | 1.766 | .329 | 28.753 | 1 | <.001 | 5.849 | 3.067 | 11.154 |
|  | DM | .780 | .258 | 9.137 | 1 | .003 | 2.181 | 1.316 | 3.617 |
|  | HT | -.595 | .277 | 4.620 | 1 | .032 | .552 | .321 | .949 |
|  | Dyslipidemia | .421 | .375 | 1.259 | 1 | .262 | 1.523 | .730 | 3.175 |
|  | MetS | .910 | .351 | 6.740 | 1 | .009 | 2.484 | 1.250 | 4.938 |
|  | Constant | -4.208 | .911 | 21.343 | 1 | <.001 | .015 |  |  |
| Step 6^a^ | site | .314 | .255 | 1.519 | 1 | .218 | 1.370 | .831 | 2.258 |
|  | Age category |  |  | 1.764 | 3 | .623 |  |  |  |
|  | age_cat(1) | -.117 | .454 | .066 | 1 | .797 | .890 | .365 | 2.167 |
|  | age_cat(2) | .171 | .452 | .144 | 1 | .705 | 1.187 | .489 | 2.879 |
|  | age_cat(3) | .344 | .504 | .466 | 1 | .495 | 1.411 | .525 | 3.787 |
|  | Sex | -.358 | .247 | 2.098 | 1 | .148 | .699 | .431 | 1.135 |
|  | Marital status |  |  | 3.903 | 2 | .142 |  |  |  |
|  | marrital_status(1) | .015 | .344 | .002 | 1 | .966 | 1.015 | .517 | 1.993 |
|  | marrital_status(2) | .677 | .450 | 2.265 | 1 | .132 | 1.967 | .815 | 4.749 |
|  | Income level |  |  | 9.493 | 4 | .050 |  |  |  |
|  | income(1) | -1.068 | .694 | 2.369 | 1 | .124 | .344 | .088 | 1.339 |
|  | income(2) | -.521 | .633 | .676 | 1 | .411 | .594 | .172 | 2.055 |
|  | income(3) | -.539 | .648 | .692 | 1 | .406 | .583 | .164 | 2.078 |
|  | income(4) | -1.287 | .660 | 3.802 | 1 | .051 | .276 | .076 | 1.007 |
|  | obesity | 1.758 | .329 | 28.579 | 1 | <.001 | 5.799 | 3.044 | 11.046 |
|  | DM | .771 | .257 | 8.972 | 1 | .003 | 2.161 | 1.305 | 3.579 |
|  | HT | -.633 | .275 | 5.322 | 1 | .021 | .531 | .310 | .909 |
|  | MetS | 1.009 | .341 | 8.734 | 1 | .003 | 2.742 | 1.405 | 5.353 |
|  | Constant | -3.857 | .847 | 20.738 | 1 | <.001 | .021 |  |  |
| Step 7^a^ | site | .277 | .252 | 1.211 | 1 | .271 | 1.319 | .805 | 2.162 |
|  | Age category |  |  | 2.079 | 3 | .556 |  |  |  |
|  | age_cat(1) | -.112 | .440 | .064 | 1 | .800 | .894 | .377 | 2.120 |
|  | age_cat(2) | .196 | .435 | .203 | 1 | .652 | 1.216 | .519 | 2.851 |
|  | age_cat(3) | .383 | .486 | .622 | 1 | .430 | 1.467 | .566 | 3.801 |
|  | Sex | -.337 | .246 | 1.883 | 1 | .170 | .714 | .441 | 1.155 |
|  | Income level |  |  | 8.828 | 4 | .066 |  |  |  |
|  | income(1) | -1.002 | .689 | 2.114 | 1 | .146 | .367 | .095 | 1.417 |
|  | income(2) | -.504 | .629 | .641 | 1 | .423 | .604 | .176 | 2.074 |
|  | income(3) | -.549 | .645 | .725 | 1 | .394 | .577 | .163 | 2.044 |
|  | income(4) | -1.257 | .657 | 3.668 | 1 | .055 | .284 | .079 | 1.030 |
|  | obesity | 1.731 | .327 | 28.003 | 1 | <.001 | 5.648 | 2.975 | 10.725 |
|  | DM | .798 | .256 | 9.718 | 1 | .002 | 2.222 | 1.345 | 3.670 |
|  | HT | -.597 | .273 | 4.769 | 1 | .029 | .551 | .322 | .941 |
|  | MetS | .965 | .339 | 8.097 | 1 | .004 | 2.625 | 1.350 | 5.105 |
|  | Constant | -3.741 | .828 | 20.409 | 1 | <.001 | .024 |  |  |
| a. Variable(s) entered on step 1: education, marrital_status, income, alcohol, current smoker, obesity, DM, HT, Dyslipidemia, MetS, highWC. | | | | | | | | | |
